# Validation of a duplex real-time PCR (qPCR) in clinically classified dogs naturally infected with *Leishmania infantum* before and after specific treatment: preliminary results

**DOI:** 10.1186/s13071-026-07490-2

**Published:** 2026-06-21

**Authors:** Clara Gómez-Velasco, Ana Montoya, Rocío Checa, Jaime Heredia-Caldeiro, Juan Pedro Barrera, Efrén Estévez-Sánchez, Juliana Sarquis, Daniela García-Rupérez, Pablo Moraleda, Guadalupe Miró

**Affiliations:** 1 Animal Health Department, Faculty of Veterinary Medicine, Complutense University of Madrid, Madrid, Spain; 2 Department of Microbiology and Parasitology, Faculty of Pharmacy, Complutense University of Madrid, Madrid, Spain

**Keywords:** Canine leishmaniosis, Polymerase chain reaction, Lymph node, Non-invasive samples, Clinical staging, Follow-up

## Abstract

**Background:**

Canine leishmaniosis, caused by *Leishmania infantum*, is a chronic vector-borne zoonosis with variable clinical manifestations, which complicates its diagnosis and monitoring. Although quantitative serology is the primary indirect diagnostic approach, polymerase chain reaction (PCR) is the most sensitive direct diagnostic method for confirming the infection by detecting *Leishmania* DNA in biological samples, but its performance varies according to the analysed tissue and molecular assay. This study aimed to validate a duplex real-time PCR assay and compare its diagnostic performance with a nested PCR in six different canine tissues, as well as to define the diagnostic value of non-invasive samples in clinical staging and monitoring in naturally infected dogs before and after specific treatment.

**Methods:**

*Leishmania* nested PCR and quantitative PCR targeting the small subunit (SSU) ribosomal RNA (rRNA) gene were performed on lymph node, blood, oral and conjunctival swabs, urine and hair samples from 66 dogs (20 non-infected, 15 clinically healthy infected and 31 naturally infected sick dogs classified according to LeishVet clinical staging). Sick dogs classified as stage II–III were re-evaluated 30 days after therapy to assess clinical improvement and changes in parasite load during follow-up.

**Results:**

Lymph node samples showed the highest sensitivity and parasite loads, correlating well with clinical severity, IFAT titres and albumin/globulin ratio. Among non-invasive samples, oral swabs showed the best performance, particularly in dogs with clinically relevant disease, while conjunctival swabs showed moderate sensitivity. By contrast, blood showed limited and stage-dependent diagnostic value, while urine and hair demonstrated very low sensitivity. Quantitative PCR showed slightly higher sensitivity than nPCR in most tissues, except for lymph node and blood samples. A *C*_T_ cut-off of 39.00 was applied to minimise non-specific amplification, which was particularly pronounced in urine qPCR, achieving 100% specificity across all other tissues. The qPCR showed a reaction efficiency of 85.4% (*R*^2^ = 0.997), with a limit of detection of 0.5 parasite equivalents/reaction. Parasite load increased significantly from stage I to stage II dogs, but did not differ between stages II and III. After treatment, clinical scores and parasite loads decreased significantly in lymph node, oral and conjunctival swab samples.

**Conclusions:**

PCR performance in canine leishmaniosis is strongly influenced by tissue, technique and clinical stage. nPCR should be the test of choice for diagnostic confirmation, with lymph node as the gold standard sample, while oral swabs represent the most reliable non-invasive alternative, particularly in dogs with clinically relevant disease. For clinical follow-up, qPCR is preferable due to its ability to monitor parasite load changes in appropriate tissues, particularly lymph node and oral swab samples. The limited reliability of PCR from non-invasive samples in early stages of infection reinforces the need for a multimodal diagnostic approach in the clinical management of canine leishmaniosis.

**Graphical Abstract:**

**Figure:**
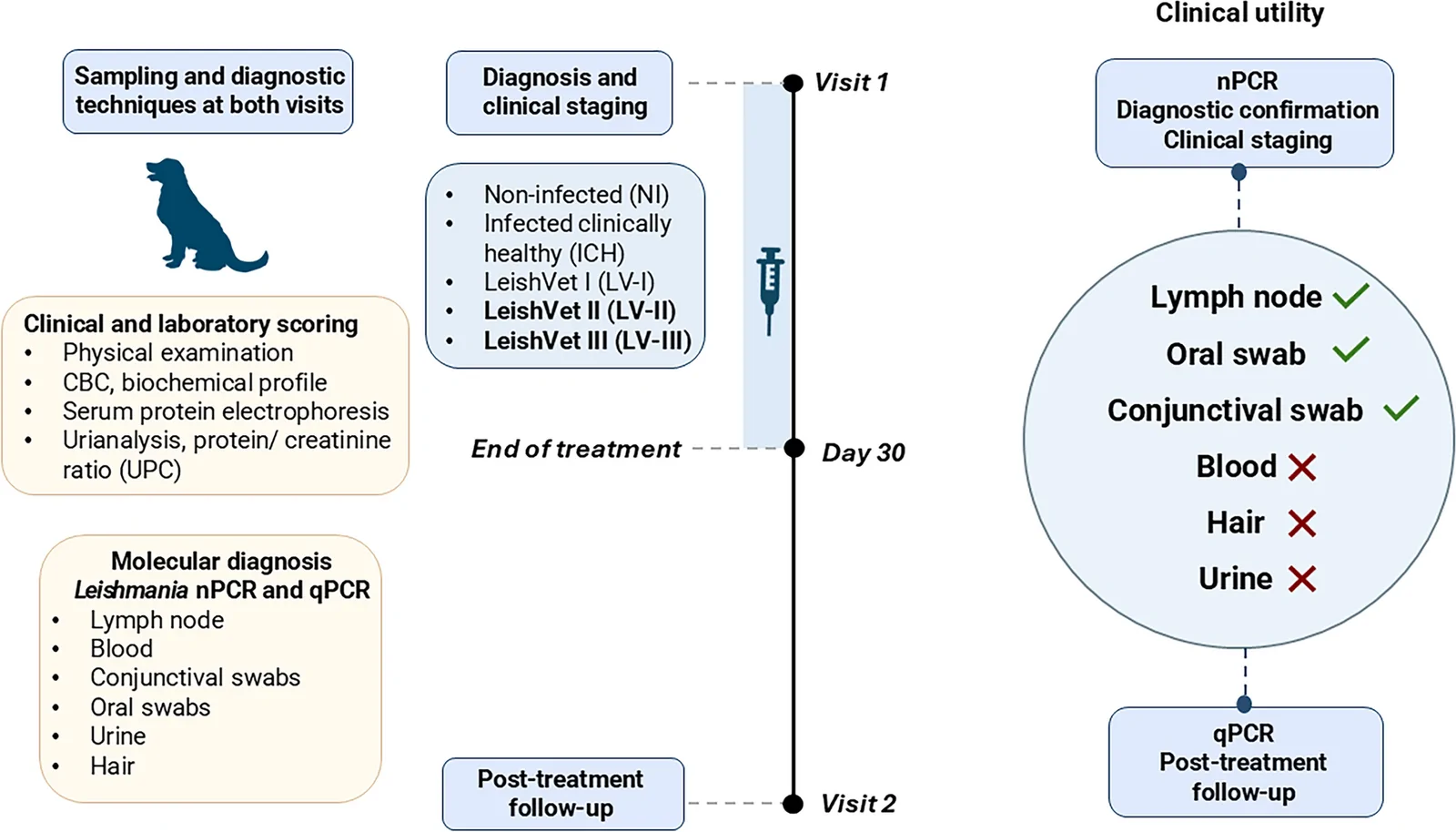

**Supplementary Information:**

The online version contains supplementary material available at https://doi.org/10.1186/s13071-026-07490-2.

## Background

Canine leishmaniosis (CanL), caused by the protozoan *Leishmania infantum*, is a widely distributed and endemic vector-borne zoonosis throughout the Mediterranean basin, the Middle East, Central and South America and several tropical and subtropical regions [1]. Dogs are considered the main peridomestic reservoir of infection [2, 3], playing an important role in its transmission to humans and other animals through blood-sucking phlebotomine sand flies.

Following infection, *Leishmania* parasites may disseminate to any organ, tissue or biological fluid. This broad tissue tropism, combined with the complex interactions between the pathogen and the host´s immune response, results in a wide spectrum of non-specific clinical signs, among which the most frequent initial manifestations are lethargy, weight loss, cutaneous lesions and lymphadenomegaly, while ocular lesions, fever, diarrhoea, epistaxis and lameness, among others, occur less commonly [1, 4]. However, because of the chronic and often insidious course of the infection, many dogs remain subclinically infected for months or even years after exposure [1, 3]. On the other hand, in those dogs that develop clinical disease, signs may improve after treatment; however, parasitological cure is rarely achieved, and relapses are frequent [5, 6], often characterised by intermittent clinical manifestations and/or clinicopathological abnormalities. Moreover, treatment response varies among sick dogs, contributing to the unpredictable clinical course of CanL and making it a significant diagnostic challenge for veterinary practitioners.

Although quantitative serology is recommended as the primary indirect diagnostic approach [1], it does not indicate active infection, nor does it directly reflect the clinical condition of the patient. Consequently, current guidelines for the prevention and control of CanL advocate a multimodal diagnostic strategy to support clinical staging and therapeutic decision-making [1, 7], consisting of clinical and clinicopathological diagnosis as well as specific laboratory tests, such as serological and parasitological techniques, including molecular diagnosis. However, clinical staging is not always easy, as discrepancies between clinical findings and other diagnostic techniques may lead to inconclusive profiles that do not fit the established classification criteria.

In this context, monitoring parasitological status during post-treatment follow-up may assist in the classification of challenging patients. Polymerase chain reaction (PCR) assays are the most sensitive direct diagnostic methods for detecting *Leishmania* DNA in biological samples and can be extremely valuable not only for initial diagnosis, but also for monitoring treatment response [8]. The sensitivity of molecular assays depends considerably on the tissue of choice. The most suitable samples for detecting *Leishmania* DNA are bone marrow, lymph node, spleen and skin [1], with bone marrow and skin potentially offering higher sensitivity and parasite loads even in subclinical infections [9, 10], which reinforces the importance of asymptomatic dogs as epidemiological reservoirs of the infection; however, their collection involves invasive procedures, restricting their applicability for repeated sampling during follow-up check-ups. Over the past decade, non-invasive samples gained prominence in the context of CanL molecular diagnosis, showing promising results [11–17]. However, their diagnostic reliability under routine clinical conditions remains uncertain, as previous studies evaluating their performance along with clinical information differ widely in clinical classification systems, type of samples and PCR protocols evaluated [17–20].

In this scenario, we hypothesised that PCR from non-invasive samples could provide clinically meaningful information when applied strategically according to tissue type, molecular technique and clinical stage, thereby complementing clinical classification of dogs according to the LeishVet staging system [1] and supporting post-treatment follow-up and clinical decision-making in CanL.

This prospective observational study is part of an ongoing longitudinal investigation in which a larger cohort of dogs will be monitored for 1 year. The results presented here aim to validate, in canine samples, a duplex real-time PCR (qPCR) assay previously developed for human specimens [21] to simultaneously detect *Leishmania* and mammalian DNA. The inclusion of a mammalian target provides an internal amplification control and may also be useful as a reference for host cellular DNA in samples with variable cellular content, such as swabs and urine. In addition, we compared its diagnostic performance with a nested PCR (nPCR) targeting the same region [small subunit (SSU) ribosomal RNA (rRNA)] to enable direct comparison between both protocols. Although kDNA-based qPCR assays are known for their high sensitivity [22], cross-amplification with other trypanosomatids has been documented [23], compromising their specificity. This limitation is overcome by the primers used in our study, which flank a region of the 18S rRNA gene specific to the *Leishmania* genus. Despite the lower copy number of this target, the use of a specific probe for the qPCR assay allows for a comparable analytical sensitivity [21, 24]. We also assessed the sensitivity of six different tissues for the detection of *Leishmania* DNA and evaluated the utility of qPCR for monitoring parasite load in non-invasive samples and its relationship with clinical staging and treatment response in naturally infected dogs before and after specific leishmanicidal treatment, to better define the diagnostic value of non-invasive samples in real-world conditions.

## Methods

### Study population and design

The study population comprised owned dogs presented to the Infectious Diseases Unit of the Veterinary Teaching Hospital, Universidad Complutense de Madrid (UCM), Spain, between September 2023 and September 2025, either after an initial diagnosis of CanL or for follow-up. Dogs were considered eligible if naturally infected with *L. infantum*, confirmed by positive serology [indirect fluorescent antibody test (IFAT cut off ≥ 1:200)] and/or a previous positive cytological or PCR diagnosis. Those who were negative at the time of enrolment could also be included, provided there was documented evidence of prior infection and a good clinical response following a first treatment course. Non-infected dogs were also enrolled as controls. Recruitment was done under routine clinical conditions, and the presence of concurrent comorbidities did not preclude inclusion, except for dogs with severe conditions likely to affect survival or interfere with clinical evaluation, including end-stage cardiac or kidney disease and/or malignant neoplasia, which were excluded from the study. In addition, dogs were not considered for enrolment if they were pregnant or lactating, had received leishmanicidal treatment within the previous 6 months, or were co-infected with other vector-borne pathogens of epidemiological relevance in Spain (*Ehrlichia canis*, *Anaplasma* spp., *Dirofilaria immitis* and *Babesia* spp.). Co-infections were assessed using rapid immunochromatographic tests (SNAP 4Dx Plus, IDEXX Laboratories, USA) and specific PCR assays for *Babesia* spp. in dogs with relevant epidemiological history and/or a clinical presentation compatible with the infection by the above-mentioned pathogens.

### Clinical evaluation and staging

At enrolment [visit 1 (V1)] and at each follow-up visit, all dogs underwent a thorough physical examination. Clinical signs were recorded and graded from 0 to 3 (absent to severe) to obtain a total clinical score with a maximum of 70 points (Additional file 1: Table S1), following a modified version of the scoring system described by Miró et al. [25]. To further assess clinical condition, routine laboratory tests were performed, including complete blood count (CBC), biochemical profile [urea, creatinine and alanine aminotransferase (ALT)], serum protein electrophoresis and *Leishmania* IFAT [26]. For the latter, serum serial dilutions ranging from 1:25 to 1:6400 were prepared, and seropositivity was defined at a cut-off titre ≥ 1:200. Urinalysis [urine specific gravity, sediment examination and protein/creatinine ratio (UPC)] was also performed at each visit. Laboratory abnormalities were graded from 0 to 3 according to severity, with a maximum score of 50 points (Additional file 2: Table S2). Each dog was assigned a total score, calculated as the sum of the clinical and laboratory scores. Finally, dogs were classified into four clinical stages (LV I–IV) according to LeishVet guidelines [1], based on their clinical history, physical examination and clinicopathological findings. Infected clinically healthy dogs (ICH) were defined as animals with a previous history of confirmed infection and showing no clinical signs or clinicopathological abnormalities compatible with CanL.

### Treatment protocol

Treatment decisions were made by clinical veterinary parasitologists, following international guidelines. ICH dogs and those classified as LV–I did not receive specific treatment and were evaluated only at V1 for the purpose of this study. Dogs in stages II and III were treated with a combination of meglumine antimoniate (MGA; 50 mg/kg subcutaneously every 12 h for 4–6 weeks) and allopurinol (10 mg/kg orally every 12 h for 6–12 months) [1]. When considered necessary, prednisone (0.5 mg/kg orally every 12 h for 3–4 weeks) was added to the initial treatment regimen in LV–III dogs to control signs associated with immune-complex deposition [27]. Proteinuric and/or azotemic dogs received supportive therapy according to the International Renal Interest Society (IRIS) guidelines for canine chronic kidney disease [28].

### Study groups and follow-up

The dog population was divided into the following groups: non-infected dogs (NI), infected clinically healthy dogs (ICH), dogs classified as LeishVet stage I (LV–I), stage II (LV–II) and stage III (LV–III). Dogs classified as LV–IV were not included in the study owing to the severity of the disease and the associated low survival rate. Follow-up included a second visit (V2), scheduled 30 days after completion of the leishmanicidal treatment.

### Sample collection

For this study, *Leishmania* qPCR and nPCR were performed on lymph node (LN), whole blood (B), conjunctival (CS) and oral swabs (OS), urine (U) and hair (H) samples collected at each scheduled visit.

Lymph node samples were obtained from the most palpable nodes (prescapular or popliteal) by gentle puncture using 20–23 G needles, depending on the dog’s size. The material was transferred into sterile tubes containing 200 µl of NET-10 preservation buffer [100 mM NaCl, 10 mM EDTA, 10 mM Tris–HCl; pH 8.0 (Thermo Fisher Scientific, Belgium)] and stored at 4 °C until processing. Exfoliative oral cells were collected by rubbing a sterile swab along the buccal mucosa and gingiva. Conjunctival samples were obtained by swabbing the lower eyelid of both eyes for 1–2 min. Hair was plucked from cutaneous lesions when present or from the inner surface of the pinna in dogs without cutaneous involvement. All swabs, urine and hair samples were stored at −20 °C until processing. Sample size varied between tissues due to differences in sample availability and collection feasibility under routine clinical conditions.

### DNA extraction

DNA was extracted from all samples using QIAamp^®^ DNA Mini Kit (QIAGEN, Germany) following the manufacturer’s instructions, with minor adaptations in the pre-treatment and elution steps for each biological matrix. Lymph node samples were resuspended in 180 µl of lysis buffer and 20 µl of proteinase K, then incubated at 56 °C until complete lysis before continuing with the standard protocol. Urine samples (2 ml) were centrifuged at 14,000 rpm for 10 min, the supernatant discarded and the DNA was obtained from the resulting sediment. Oral and conjunctival swabs were cut from the sticks using sterile scalpels, immersed in 400 µl of sterile 1× phosphate-buffered saline (PBS) (Roche Diagnostics GmbH, Germany) and incubated with 20 µl of proteinase K and 400 µl of lysis buffer. The swab material was then compressed to maximise liquid phase recovery, yielding a final volume of 1 ml. Hair samples (10–15 hairs) were incubated in 300 µl of lysis buffer, 20 µl of proteinase K and 20 µl of 1 M 1.4-Dithiothreitol (DTT) (Roche Diagnostics GmbH, Germany) until complete lysis. All DNA extracts were stored at −20 °C until PCR amplification.

### Nested PCR (nPCR)

A nested PCR assay targeting the variable region of the small subunit ribosomal RNA (SSU rRNA) gene was performed to detect *Leishmania* spp. DNA, following a protocol modified from Cruz et al. [24]. The method consists of two successive amplification rounds to enhance detection sensitivity and specificity.

The primers used were R1 (R221) (5′-GGTTCCTTTCCTGATTTACG-3′) and R2 (R332) (5′-GGCCGGTAAAGGCCGAATAG-3′), specific for order *Kinetoplastida* [29], for the first reaction, and R3 (R223) (5′-TCCCATCGCAACCTCGGTT-3′) and R4 (R333) (5′-AAAGCGGGCGCGGTGCTG-3′), specific for the genus *Leishmania* spp. [29], for the second amplification.

For the first amplification, 20 µl of extracted DNA were added to 30 µl of a master mix containing 15 pmol of each primer (R1 and R2), 5 µl of 10× buffer, 2 mM of MgCl_2_, 1 µl of 10 mM dNTPs and 1.4 U of *Tth* DNA polymerase (1 U/µl) (Biotools, Spain). The reaction was performed in a GenAmp^®^ PCR System 2700 thermocycler (Applied Biosystems, Spain) under the following cycling conditions: initial denaturation at 80 °C for 2 min and 94 °C for 5 min, followed by 30 cycles of: 94 °C for 30 s, 60 °C for 30 s and a final extension at 72 °C for 10 min. For the second amplification, the first-round PCR product was diluted 1:40 in sterile distilled water and 10 µl of this dilution were added to 15 µl of a master mix containing 7.5 pmol of primer R3, 3.75 pmol of primer R4, 2.5 µl of 10× buffer, 2.0 mM of MgCl_2_, 0.5 µl of 10 mM dNTPs and 0.7 U of *Tth* DNA polymerase (1 U/µl) (Biotools, Spain). Cycling conditions were: 80 °C for 2 min, 94 °C for 5 min, followed by 30 cycles of: 94 °C for 30 s, 65 °C for 30 s and 72 °C for 30 s with a final extension at 72 °C for 5 min and hold at 4 °C. PCR products from the second reaction were subjected to electrophoresis on 1.5% agarose gels (D1 Low EEO) prepared in 1× TAE buffer (Roche Diagnostics GmbH, Germany) and then stained with SYBR™ Safe DNA Gel Stain (Invitrogen, Spain) and visualized under a Bio-Rad transilluminator. Samples showing a specific band of the expected size (353 bp) were considered positive for *Leishmania* spp. DNA.

### Quantitative PCR (qPCR)

A duplex real-time PCR (qPCR) protocol, described by Chicharro et al. [21], was used for the simultaneous detection of *Leishmania* spp. and mammalian DNA. *Leishmania* detection employed primers from the second nPCR reaction (R3 and R4, described above) [24], while mammalian DNA amplification was based on the primers reported by Norman et al. [30]: HUF (5′-GAGCCGCCTGGATACCGC-3′) and REV (5′-GACGGTATCTGATCGTCTTC-3′). The mammalian target was used exclusively as an internal amplification control to confirm the presence of amplifiable DNA and to detect potential PCR inhibition. Amplifications were performed on a Corbett Rotor-Gene™ 6000 real-time PCR System (Qiagen) following the cycling conditions described in Chicharro et al. [21]. Samples were considered positive for *Leishmania* spp. when *C*_T_ values were below the established cut-off (*C*_T_ < 39). All samples were analysed in duplicate, with each assay including two positive controls, a blank extraction control (BEC) and a no-template control (NTC) using sterile water to monitor contamination throughout the process. The same set of controls was applied to both *Leishmania* and mammalian targets, ensuring consistent and reliable performance of the duplex assay.

#### Standard curve

A standard curve was generated using tenfold serial dilutions of *L. infantum* DNA spiked into a canine DNA solution obtained from an uninfected healthy dog. Five dilutions were amplified in duplicate, ranging from 5 × 10^5^ parasite equivalents/ml. Parasite load was quantified on the basis of *Leishmania C*_T_ values and expressed as parasite equivalents/ml following the equation *y* = −3.731*x* + 40.037. Although efficiency was slightly lower than ideal (85.4%), potentially reflecting minor variability associated with standard curve preparation and amplification conditions, the standard curve showed excellent linearity (*R*^2^ = 0.997), ensuring the reliability of the assay. This may introduce certain variability in parasite load quantification in samples with low parasite burdens or in the presence of PCR inhibitors. The equation was calculated using *C*_T_ values with a coefficient of variation below 25% (Fig. 2a). The limit of detection (LOD) was defined as the lowest standard concentration yielding consistent amplification in both duplicates, corresponding to 50 parasites/ml (0.5 parasite equivalents/reaction), which is comparable to values previously reported for this qPCR protocol in human samples [21].

### Statistical analysis

All statistical analyses were performed using the software package SPSS Statistics version 28 (SPSS Inc., Chicago, II, USA). Graphs were created using GraphPad Prism 10 (GraphPad software, Boston, Massachusetts, United States). Non-parametric tests were applied after assessing data normality using the Shapiro–Wilk test. Group comparisons for clinical variables and qPCR parasite loads were performed using the Kruskal–Wallis test (*H*) with Bonferroni correction. nPCR results were analysed using the Fisher–Freeman–Halton exact test, with adjusted residuals (*z*) used to assess group discriminatory capacity of each tissue and technique. Cells with absolute *z* > 1.96 were considered statistically significant. Agreement between PCR techniques and tissues was assessed using Cohen’s kappa coefficient (*κ*). Kappa values were interpreted according to standard thresholds: *κ* < 0.20, slight; *κ* = 0.21–0.40, fair; *κ* = 0.41–0.60, moderate; *κ* = 0.61–0.80, substantial; and *κ* = 0.81–1.00, almost perfect agreement. Paired qualitative comparisons were performed using McNemar’s test. Correlations between continuous variables were evaluated using Spearman’s rank coefficient (*r*_s_) and were interpreted as: *r*_s_ = 0–0.19, very weak; *r*_s_ = 0.20–0.39, weak; *r*_s_ = 0.40–0.59, moderate; *r*_s_ = 0.60–0.79, strong; and *r*_s_ = 0.80–1.00, very strong. Longitudinal changes between visits were analysed using the Wilcoxon signed-rank test (*Z*), and differences in parasite load reduction between LV–II and LV–III dogs were assessed using the Mann–Whitney *U* test. Statistical significance was set at *P* < 0.05. Receiver operating characteristic (ROC) curve analysis was performed to determine the *C*_T_ cut-off value for qPCR positivity, using clinical classification as the reference standard. The optimal cut-off based on Youden’s index (*J* = sensitivity + specificity − 1) was calculated for each tissue, showing moderate to good performance with the Area Under the Curve (AUC) values ranging from 0.57 (urine) to 0.81 (lymph node). However, ROC-based thresholds were considered suboptimal for clinical interpretation (*C*_T_ = 44–45), likely influenced by the inclusion of infected clinically healthy and LeishVet stage I dogs, with low parasite loads. Therefore, the final C_T_ cut-off (*C*_T_ < 39) was defined by integrating the analytical performance of the ROC curves with clinical plausibility and comparability with previously reported values for this qPCR protocol [21].

## Results

### Study dog population

Of the 66 dogs included in the study, 36 were male and 30 were female. In total, 44 (66.7%) were neutered and 22 (33.3%) remained intact. On the basis of age, six dogs were classified as junior (9 months–2 years), 14 as young adults (2–4 years), 30 as mature adults (5–8 years) and 16 as senior (>8 years). The mean age was 6 years (range: 9 months to 15 years). Overall, 27 dogs were mongrels and 39 were purebred. Among the purebred dogs, the most represented breeds were Beagle (33.3%), Teckel (10.3%), Spanish Greyhound (7.7%) and French Bulldog (7.7%). The distribution of dogs across clinical categories was as follows: 20 NI, 15 ICH, 7 LV–I, 14 LV–II and 10 LV–III.

### Clinical, laboratory and serological parameters at visit 1

**Fig. 1:**
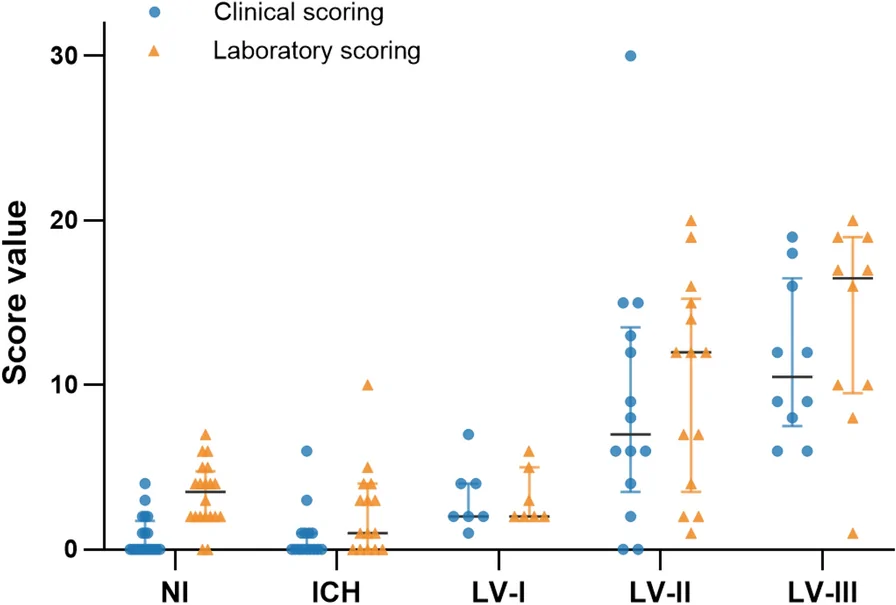
Clinical and laboratory scores across dog groups at visit 1. Individual clinical scores (blue dots) and laboratory scores (orange triangles) are shown for non-infected (NI), infected clinically healthy (ICH) and LeishVet stages I–III (LV–I, LV–II, LV–III) dogs. Horizontal grey lines indicate median values and whiskers represent interquartile ranges. Clinical and laboratory scores were based on the grading systems described in Additional file 1: Table S1 (maximum of 70 points) and Additional file 1: Table S2 (maximum of 50 points), respectively. The clear increase in both scores from NI and ICH dogs to LV–II and LV–III dogs reflects the clinical and laboratory changes expected across disease stages

Baseline clinical, laboratory and serological data at V1 are summarised in Additional file 3. Both clinical (CLS) and laboratory scores (LS) showed a clear progressive increase from NI and ICH dogs to those classified as LV–II and LV–III (Fig. 1). NI and ICH dogs exhibited minimal clinical and laboratory alterations, with low mean scores (CLS: NI 0.80 ± 1.20, ICH 0.87 ± 1.64; LS: NI 3.30 ± 1.92, ICH 2.33 ± 2.74), as well as LV–I dogs, that showed mild increases (CLS: 3.14 ± 2.04; LS: 3.14 ± 1.68), consistent with early stages of the disease and limited systemic involvement. By contrast, LV–II (CLS: 9.00 ± 7.85; LS: 10.21 ± 6.40) and LV–III dogs (CLS: 11.50 ± 4.77; LS: 13.70 ± 6.18) displayed markedly higher clinical and laboratory scores, with no significant differences between these two groups. Systemic clinical signs were uncommon in NI, ICH and LV–I groups but were observed in the majority of LV–II (71.4%) and in all LV–III dogs (100.0%), as well as cutaneous manifestations. Additionally, signs compatible with immune-complex deposition were present in most LV–III dogs (70.0%). Dogs at stages II and III predominantly showed medium to high IFAT titres (1/800–1/6400), while ICH and LV–I dogs only had negative to low positive titres (≤ 1/400). NI dogs were uniformly seronegative (< 1/200).

### Diagnostic performance of nPCR and qPCR on clinically classified dogs

#### PCR positivity by tissue and clinical group

At V1, PCR positivity in both techniques varied markedly by tissue and clinical group (Table 1). Lymph node samples showed the highest detection rates, with a clear increase from ICH and LV–I dogs to LV–II and LV–III, reaching 81.8–100% in advanced stages. Conjunctival and oral swabs followed a similar pattern, with positivity rising in stages II–III (50.0–83.3%), while detection in early stages was minimal or absent, particularly with nPCR. Accordingly, PCR positivity for all these tissues differed significantly across groups (Fisher–Freeman–Halton exact test, *P* < 0.001), with over-representation of positive results in LV–II and LV–III dogs compared with ICH and LV–I dogs, for both nPCR (LN *z* = 3.9; CS *z* = 4.0; OS *z* = 4.8 in LV–II, and LN *z* = 4.3; CS *z* = 2.6; OS *z* = 3.3 in LV–III) and similarly for qPCR (data not shown). Blood samples showed intermediate detection rates, with low positivity in early stages (0.0–28.6% of ICH and LV–I dogs) and higher values in advanced stages. nPCR detected *Leishmania* DNA more frequently than qPCR in LV II–III dogs (50.0–60.0% with nPCR versus 28.6–50.0% with qPCR). For this tissue, positivity also differed significantly across groups (Fisher–Freeman–Halton exact test, *P* < 0.001), mainly driven by over-representation of positive results in LV–III dogs for both techniques (nPCR *z* = 3.3; qPCR *z* = 3.1), and in LV–II dogs only for nPCR (*z* = 3.0), suggesting a stage-dependent association of blood positivity with more advanced disease. Urine and hair samples exhibited the lowest positivity rates and weakest associations with the studied groups. nPCR obtained only sporadic positive results, limited to LV–II for U (1/9), and LV–III for H (3/10). qPCR detection in urine was also low, with sporadic positives across different groups and no clear clinical pattern. Group differences were statistically significant only for H nPCR (Fisher–Freeman–Halton exact test, *P* = 0.018). In the same way, no significant differences in positivity rates were found between ICH and LV–I or between LV–II and LV–III dogs for any tissue or PCR protocol (data not shown).

**Table 1:** nPCR and qPCR positivity by tissue and clinical group

	NI (*n* = 20)		ICH (*n* = 15)		LV–I (*n* = 7)		LV–II (*n* = 14)		LV–III (*n* = 10)		Total (*n* = 66)		*P* value (across groups)
	Pos/*n*	% (95% CI)	Pos/*n*	% (95% CI)	Pos/*n*	% (95% CI)	Pos/*n*	% (95% CI)	Pos/*n*	% (95% CI)	Pos/*n*	% (95% CI)	
LN													
nPCR	0/19	0.0	3/14	21.4 (6.4–46.9)	1/7	14.3 (1.6–50.1)	10/11	90.9 (64.7–99.0)	10/10	100.0	24/61	39.3 (27.8–51.9)	< 0.001*
qPCR	0/19	0.0	3/14	21.4 (6.4–46.9)	1/7	14.3 (1.6–50.1)	9/11	81.8 (48.2–97.7)	9/10	90.0 (55.5–99.7)	22/61	36.1 (24.2–49.4)	< 0.001*
CS													
nPCR	0/20	0.0	0/15	0.0	0/7	0.0	8/14	57.1 (31.9–79.7)	5/10	50.0 (22.4–77.7)	13/66	19.7 (11.5–30.5)	< 0.001*
qPCR	0/20	0.0	1/15	6.7 (0.2–31.9)	2/7	28.6 (6.5–64.8)	8/14	57.1 (31.9–79.7)	5/10	50.0 (22.4–77.7)	16/66	24.2 (14.5–36-4)	< 0.001*
OS													
nPCR	0/20	0.0	0/13	0.0	0/7	0.0	10/12	83.3 (56.4–96.4)	7/10	70.0 (39.4–90.7)	17/62	27.4 (17.5–39.4)	< 0.001*
qPCR	0/20	0.0	0/13	0.0	1/7	14.3 (1.6–50.1)	10/12	83.3 (51.6–97.9)	6/10	60.0 (30.4–84.7)	17/62	27.4 (17.5–39.4)	< 0.001*
B													
nPCR	0/20	0.0	0/15	0.0	1/7	14.3 (1.6–50.1)	7/14	50.0 (25.9–74.1)	6/10	60.0 (30.4–84.7)	14/66	21.2 (12.7–32.2)	< 0.001*
qPCR	0/20	0.0	0/15	0.0	2/7	28.6 (6.5–64.8)	4/14	28.6 (10.5–54.5)	5/10	50.0 (18.7–81.3)	11/66	16.7 (8.6–27.9)	< 0.001*
U													
nPCR	0/7	0.0	0/13	0.0	0/7	0.0	1/9	11.1 (1.2–41.5)	0/8	0.0	1/44	2.3 (0.3–10.1)	0.705
qPCR	1/7	14.3 (0.4–57.9)	3/13	23.1 (7.0–49.7)	0/7	0.0	0/9	0.0	1/8	12.5 (1.38–45.4)	5/44	11.4 (3.8–24.6)	0.503
H													
nPCR	0/20	0.0	0/14	0.0	0/7	0.0	2/14	14.3 (3.1–38.5)	3/10	30.0 (9.3–60.6)	5/65	7.7 (3.0–16.0)	0.018*

Sample size varies between tissues owing to differences in sample availability

*nPCR* nested PCR, *qPCR* quantitative PCR, *NI* non-infected dogs, *ICH* infected clinically healthy dogs, *LV I–III* dogs classified as LeishVet stages I–III, *n* number of dogs, *Pos/n* positive samples/number of dogs, *CI* confidence interval, *LN* lymph node, *CS* conjunctival swabs, *OS* oral swabs, *B* blood, *U* urine, *H* hair

Asterisks (*) indicate statistically significant differences (*P* < 0.05)

#### Sensitivity and specificity of PCR techniques

Sensitivity and specificity were calculated using clinical classification as the reference standard (Table 2). Overall, qPCR showed slightly higher sensitivity than nPCR in most tissues, except for lymph node and blood samples, where nPCR performed better (LN: 57.1% versus 52.4%; B: 30.4% versus 23.9%), while specificity was consistently high (100%) for both methods, except for qPCR in urine (85.7%). Among tissues, LN showed the highest diagnostic performance for both techniques and oral and conjunctival swabs showed moderate sensitivities (OS: 40.5% and CS: 28.3–34.8%). Blood samples demonstrated more limited performance, and urine and hair samples showed minimal sensitivity (< 15%).

**Table 2:** Sensitivity (Se), specificity (Sp) and agreement (*κ*) between nPCR and qPCR by tissue

	Se (%)	Sp (%)	*κ* value between techniques
LN			
nPCR	57.1	100.0	0.86
qPCR	52.4	100.0	
CS			
nPCR	28.3	100.0	0.69
qPCR	34.8	100.0	
OS			
nPCR	40.5	100.0	0.76
qPCR	40.5	100.0	
B			
nPCR	30.4	100.0	0.75
qPCR	23.9	100.0	
U			
nPCR	2.7	100.0	−0.04
qPCR	10.8	85.7	
H			
nPCR	11.1	100.0	ND

*Se* sensitivity, *Sp* specificity, *κ* Cohen’s kappa coefficient, *nPCR* nested PCR, *qPCR* quantitative PCR, *LN* lymph node, *CS* conjunctival swabs, *OS* oral swabs, *B* blood, *U* urine, *H* hair, *ND* not determined

#### Agreement analyses between diagnostic methods and tissues

Agreement between nPCR and qPCR was almost perfect for LN (*κ* = 0.86) and substantial for OS (*κ* = 0.76), CS and B (*κ* = 0.69–0.75, respectively) (Table 2). When comparing tissues with LN (Table 3), OS showed the highest agreement among all non-invasive tissues for both PCR techniques (*κ* = 0.62 for nPCR; *κ* = 0.68 for qPCR), although this was mainly driven by positive results in sick dogs (LeishVet II–III), with very limited detection in dogs in earlier stages. Conjunctival swabs and blood obtained fair to moderate agreement, with slightly higher values observed for nPCR than for qPCR (CS: 0.27–0.51; B: 0.35–0.51). Urine and hair samples displayed only slight agreement with the reference tissue (*κ* = 0.06 and 0.19, respectively).

**Table 3:** Agreement (*κ*) between lymph node and non-invasive samples for nPCR and qPCR

Result	CS		OS		B		U		H	
	Positive	Negative	Positive	Negative	Positive	Negative	Positive	Negative	Positive	Negative
*nPCR*										
LN nPCR										
Positive	12	12	14	9	12	12	1	18	4	20
Negative	1	36	1	35	1	36	0	23	0	36
*κ*-value	0.51	0.62	0.51	0.06	0.19					
*qPCR*										
LN qPCR										
Positive	9	13	14	7	8	14	0	17	ND	
Negative	6	33	1	37	2	37	5	20	ND	
*κ*-value	0.27	0.68	0.35	−0.23	ND					

*κ* Cohen’s kappa coefficient, *nPCR* nested PCR, *qPCR* quantitative PCR, *LN* lymph node, *CS* conjunctival swabs, *OS* oral swabs, *B* blood, *U* urine, *H* hair, *ND* not determined

### Parasite load in clinically classified dogs

**Fig. 2:**
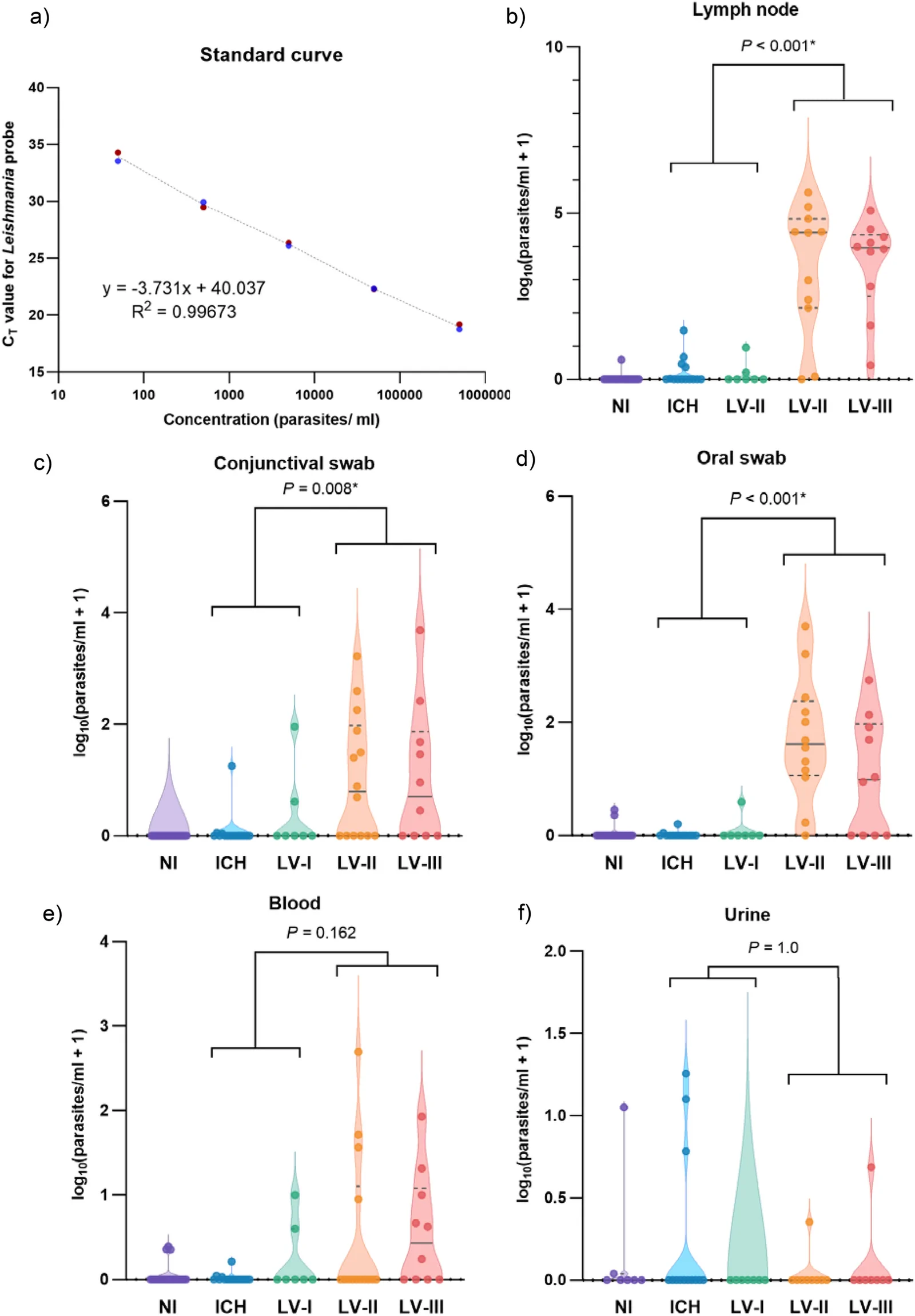
Parasite load by tissue and clinical group at visit 1. **a** Standard curve built by linear regression used for parasite quantification by qPCR. Parasite loads in lymph node (**b**), conjunctival and oral swabs (**c**, **d**), blood (**e**) and urine (**f**) are expressed as log_10_(parasites/ml + 1). Each dot represents an individual dog, and violin plots show the distribution of values within each clinical group. Horizontal lines indicate medians and interquartile ranges (IQR). Higher parasite loads were mainly observed in LV–II and LV–III dogs, particularly in lymph node and swab samples. Brackets indicate pairwise comparisons between grouped clinical categories (ICH + LV–I versus LV–II + LV–III). *P* values were calculated using Kruskal–Wallis test with Bonferroni correction and asterisks (*) indicate statistically significant differences (*P* < 0.05). NI: non-infected dogs; ICH: infected clinically healthy dogs; LV I–III: dogs classified as LeishVet stages I–III

Parasite loads measured by qPCR differed significantly among groups for LN (*H* = 41.83, *df* = 4, *P* < 0.001), CS (*H* = 20.29, *df* = 4, *P* < 0.001) and OS samples (*H* = 33.83, *df* = 4, *P* < 0.001), but not for B or U (Fig. 2). Lymph node showed the highest parasite burdens of all tissues (max. 415,207.23 parasites/ml), especially in LV–II and LV–III dogs (median 9777.23 parasites/ml, range 248.16–27,613.20), and with negligible or undetectable values in ICH and LV–I dogs (median 0.00 parasites/ml, range 0.00–0.30). Similar patterns were observed in OS and CS, but with lower parasite loads than those found in LN samples from LV II–III dogs (OS: median 27.02 parasites/ml, range 0.70–134.62; CS: median 5.33 parasites/ml, range 0.00–61.65). Parasite load in those three tissues significantly differentiated ICH and LV–I dogs from LV II–III dogs (LN: *Z* = −4.97, Bonferroni correction *P* < 0.001; CS: *Z* = −2.99, *P* = 0.008; OS: *Z* = −4.69, *P* < 0.001), but no differences were observed between LV–II and LV–III, nor between ICH and LV–I dogs. Blood samples generally showed lower parasite loads (LV II–III median 0.00, range 0.00–8.50 parasites/ml) than those found in OS and CS, with a maximum of 84.04 parasites/ml found in LV–II, and 495.87 parasites/ml in LV–III. Urine parasite loads were the lowest reported (max 16.99 parasites/ml in an ICH dog). The distribution of *C*_T_ values across clinical groups, consistent with the parasite loads described above, is summarised in Additional file 4: Table S4. qPCR results from hair samples were excluded from the analysis owing to unreliable amplification patterns, which are addressed below.

#### Parasite load correlation with clinical and laboratory parameters

Parasite load measured in LN and OS showed consistent correlations with clinical and laboratory parameters (Table 4). Lymph node parasite load correlated strongly with IFAT (*r*_s_ = 0.75, *P* < 0.001) and albumin/globulin ratio (*r*_s_ = −0.64, *P* < 0.001). Among the non-invasive tissues evaluated, OS obtained the highest positive correlation with LN parasite load (*r*_s_ = 0.60, *P* < 0.001) and had a strong correlation with IFAT (*r*_s_ = 0.62, *P* < 0.001). Correlations with clinical status (total score) were moderate for both LN (*r*_s_ = 0.57, *P* < 0.001) and OS (*r*_s_ = 0.55, *P* < 0.001).

**Table 4:** Spearman correlation coefficient (*r*_s_) between parasite load measured by qPCR from different tissues and clinical parameters

	LN qPCR	CS qPCR	OS qPCR	B qPCR	U qPCR
LN qPCR	1.00	0.42	0.60	0.37	−0.13
Total score	0.57	0.50	0.55	0.35	−0.10
IFAT	0.75	0.50	0.62	0.33	−0.18
Ratio Alb/Glob	−0.64	−0.53	−0.55	−0.33	0.25

*LN* lymph node, *CS* conjunctival swabs, *OS* oral swabs, *B* blood, *U* urine, *nPCR* nested PCR, *qPCR* quantitative PCR, *IFAT* indirect fluorescent antibody test, *Alb/Glob* albumin/globulins ratio

### PCR in assessing treatment response

For this part of the analysis, only dogs classified as LV–II and LV–III at V1 were included, as they received specific treatment and therefore had available data from both V1 and V2. Following treatment, total clinical scores decreased significantly from 21.7 ± 11.0 points to a mean of 10.1 ± 8.0 points (*Z* = 10.00, *P* < 0.001). Both LV–II and LV–III dogs showed significant improvement in clinical scores (CLS) (LV–II: *Z* = −2.94, *P* = 0.003; LV–III: *Z* = −2.65, *P* = 0.008), whereas only LV–II dogs exhibited a significant reduction in laboratory scores (LS) (*Z* = −2.77, *P* = 0.006). IFAT titres showed no significant change between both visits (*Z* = 52.50, *P* = 0.150).

**Fig. 3:**
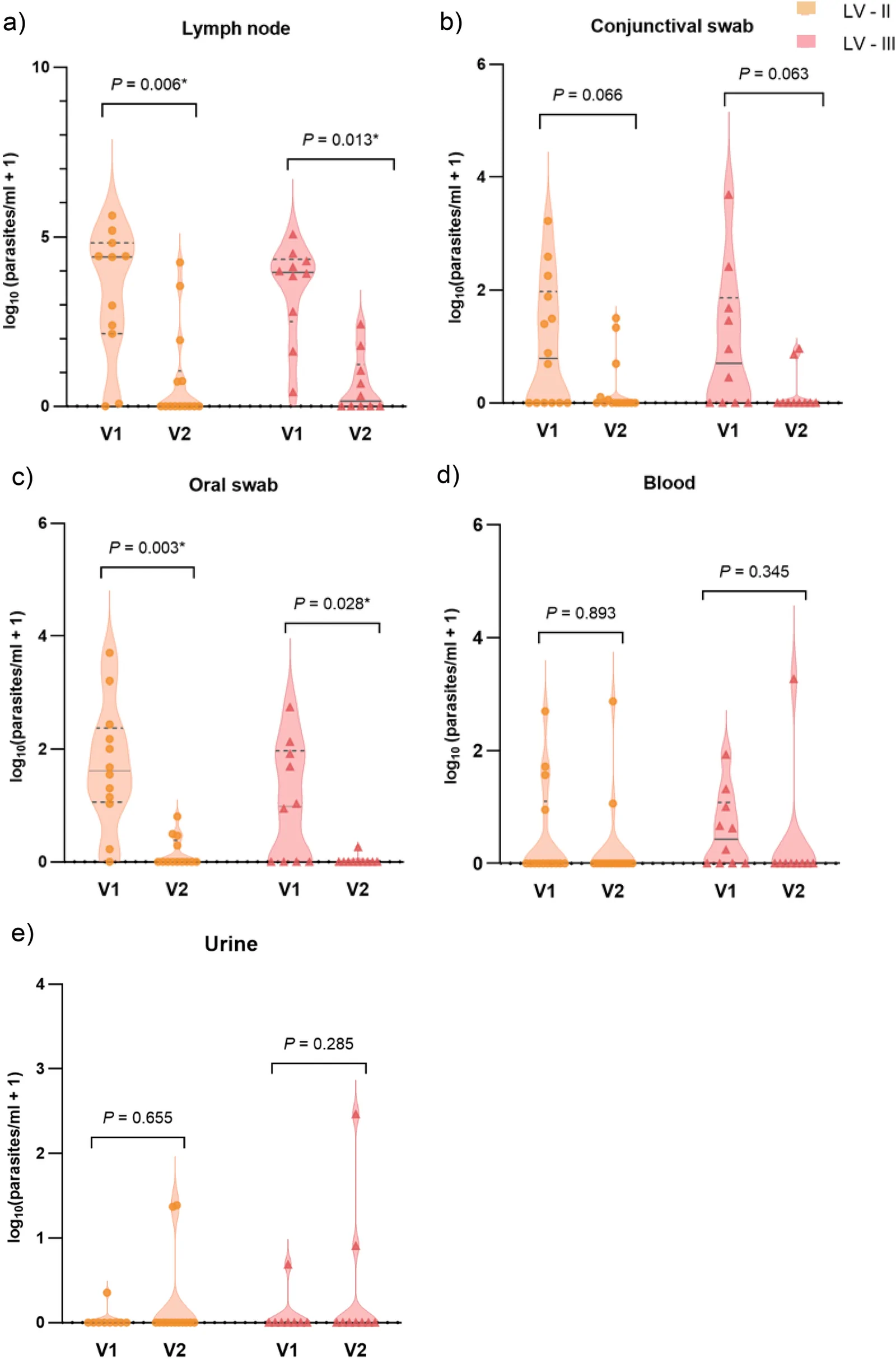
Changes in parasite load before (V1) and after treatment (V2) in LeishVet stage II and III dogs across different tissues. Parasite load quantified by qPCR [expressed as log_10_(parasites/ml + 1)] in lymph node (**a**), conjunctival and oral swabs (**b**, **c**), blood (**d**) and urine (**e**) samples at visit 1 (V1) and 30 days after completion of treatment (visit 2, V2). Results are shown for dogs classified as LeishVet stage II (LV–II; orange left violins), and stage III (LV–III; red right violins). Each point represents an individual dog, overlaid on violin plots showing the distribution and density of values. Horizontal lines indicate medians and interquartile range (IQR). Brackets indicate within-stage paired comparisons between V1 and V2. *P* values were calculated using Wilcoxon signed-rank test and asterisks (*) indicate statistically significant differences (*P* < 0.05). A general decrease in parasite load after treatment was observed, particularly in lymph node and swab samples, while changes in blood and urine parasite load were not statistically significant

At V2, nPCR positivity decreased significantly across most tissues when compared with V1, except for U and H samples, which already exhibited low positivity at baseline (Table 5). Lymph node positivity decreased from 95.2% (20/21) to 42.9% (9/21) [McNemar test (exact), *P* < 0.001], and OS went from 77.3% (17/22) to 9.1% (2/22) after treatment [McNemar test (exact), *P* < 0.001]. Conjunctival swabs and blood samples also experienced significant reductions in positivity rate. Similarly, parasite loads decreased significantly after treatment (Fig. 3). The tissue with the most pronounced reduction was the LN, with median parasite loads decreasing from 25,788.00 parasites/ml (range 139.27–67,835.95) to 0.00 parasites/ml (range 0.00–4.52) in LV–II (*Z* = 2.00, *P* = 0.006), and from 9069.00 parasites/ml (range 620.36–19,500.09) to 0.50 parasites/ml (range 0.00–10.41) in LV–III dogs (*Z* = 3.00, *P* = 0.013). Oral swabs (*Z* = 2.00, *P* < 0.001) and CS (*Z* = 18.00, *P* = 0.010) also experienced substantial decreases, while B and U samples did not show meaningful changes after treatment. No statistically significant differences in parasite load reduction were found between LV–II and LV–III dogs in any tissue.

**Table 5:** nPCR positivity by tissue at visit 1 and visit 2 in dogs with LeishVet stages II and III

Tissue	Visit	LV–II		LV–III		Total	
		% (Pos/*n*)	*P* value between visits	% (Pos/*n*)	*P* value between visits	% (Pos/*n*)	*P* value between visits
LN	V1	90.9 (10/11)	0.063	100 (10/10)	0.031*	95.2 (20/21)	< 0.001*
	V2	45.5 (5/11)		40.0 (4/10)		42.9 (9/21)	
CS	V1	57.1 (8/14)	0.016*	50.0 (5/10)	0.375	54.2 (13/24)	0.006*
	V2	7.1 (1/14)		20.0 (2/10)		12.5 (3/24)	
OS	V1	83.3 (10/12)	0.004*	70.0 (7/10)	0.031*	77.3 (17/22)	< 0.001*
	V2	8.3 (1/12)		10.0 (1/10)		9.1 (2/22)	
B	V1	50.0 (7/14)	0.219	60.0 (6/10)	0.063	54.2 (13/24)	0.012*
	V2	21.4 (3/14)		10.0 (1/10)		16.7 (4/24)	
U	V1	11.1 (1/9)	1.000	0.0 (0/8)	1.000	5.9 (1/17)	1.000
	V2	22.2 (2/9)		0.0 (0/8)		11.8 (2/17)	
H	V1	14.3 (2/14)	ND	30.0 (3/10)	ND	20.8 (5/24)	ND
	V2	0.0 (0/14)		0.0 (0/10)		0.0 (0/24)	

Comparisons between V1 and V2 were performed using McNemar’s test

*ND* not determined owing to insufficient number of paired positive results. *nPCR* nested PCR, *LV II–III* dogs classified as LeishVet stages II–III, *n* number of dogs, *Pos/n* positive samples/number of dogs, *LN* lymph node, *CS* conjunctival swabs, *OS* oral swabs, *B* blood, *U* urine, *H* hair

Asterisks (*) indicate statistically significant differences (*P* < 0.05)

## Discussion

This study evaluated the diagnostic performance of two PCR assays (qPCR and nPCR) across various tissues in dogs naturally infected with *L. infantum* and assessed their utility for clinical staging and monitoring treatment response in CanL.

Consistent with previous reports [5, 31], lymph node samples were the most sensitive matrix for the detection of *Leishmania* infection by both PCR protocols, exhibiting the highest parasite loads in our study. Lymphoid organs, such as lymph nodes and bone marrow, are well established as the preferred sampling sources for CanL molecular diagnosis, as they are target organs for parasite persistence and multiplication [32]. However, despite its high positivity rate from LV–II onwards (>80%), fewer than 30% of ICH and LV–I dogs were PCR positive in lymph node samples, highlighting the strong influence of clinical stage on PCR performance. This may be explained by the low parasite burdens found in early disease stages and/or the practical limitations of lymph node sampling, particularly in the absence of lymphadenomegaly, as it requires trained personnel and limits its applicability for repeated or large-scale sampling. These limitations reinforce the need for alternative, less invasive sampling approaches.

Among non-invasive samples, oral and conjunctival swabs showed the highest detection rates and parasite loads in our study, particularly in dogs with moderate to severe disease (LV–II and LV–III). Although their sensitivity and parasite loads were lower than those observed in lymph node samples, positive results from these matrices likely reflect systemic dissemination of the infection, as mucous membranes contain immune cell populations that may harbour *Leishmania* amastigotes reaching these locations via lymphatic or haematogenous routes [33]. Oral swabs showed higher sensitivity and parasite loads than conjunctival swabs, as previously reported in an experimental infection study [12], possibly due to the larger sampling surface, which increases the cellular yield. Furthermore, the muzzle is a frequently reported site for sand fly feeding because phlebotomine are strongly attracted to respiratory carbon dioxide gradients, leading to preferential biting of the nasal areas, and also likely owing to the absence of hair [34]. While studies have reported variable performance of oral swabs, ranging from sensitivities comparable to invasive tissues such as bone marrow [12, 13] to lower detection rates [35], conjunctival swabs have more consistently shown high positivity in both symptomatic and asymptomatic dogs [10, 14, 17, 36], with parasite loads equivalent to those observed in bone marrow regardless of clinical status [10]. This, together with their non-invasive nature, facilitates their application as a mass-screening tool in large epidemiological surveys [37]. In our study, however, swab positivity was strongly influenced by clinical stage, with very low detection in early stages, which would limit the applicability of this protocol for identifying infection in asymptomatic populations. These discrepancies between studies may be partly explained by variability in cellular yield inherent to swab sampling [36]. In this context, normalisation of parasite load to host cell content would help standardise comparisons across samples and time points, and could be incorporated in future longitudinal studies, as this qPCR assay allows for such an approach.

Overall, our results indicate that the diagnostic performance of PCR assays is strongly influenced by clinical stage, with both positivity rates and parasite loads increasing with disease severity, which effectively distinguished healthy or subclinical dogs from those with clinically relevant disease. While lymph node PCR occasionally detected infection in early stages owing to its high sensitivity, swab positivity appeared to reflect the need for wider dissemination and higher tissue parasite loads, with nPCR positivity observed only from LV–II onwards.

Parasite loads showed clear associations with clinical and laboratory markers of disease severity and prognosis in CanL. Lymph node parasite load correlated relatively well with IFAT, total score and albumin/globulin ratio, a parameter widely associated with disease progression and prognosis in CanL [38]. Oral swab parasite loads also showed moderate correlation with LN burden and serological response. These associations are consistent with previous studies linking parasite load in different biological samples with disease severity in naturally infected dogs [39, 40]. Interestingly, parasite burden from these tissues significantly increased at the transition from LV–I to LV–II but did not differ between LV–II and LV–III dogs. This finding suggests that progression from LV–II to LV–III may be more related to immunopathological mechanisms, such as immune complex deposition in target organs (e.g. kidneys or intraocular tissues), which are not accessible through routine sampling techniques. Consequently, qPCR appears to be more informative for identifying the onset of clinically relevant disease than for discriminating between advanced clinical stages. In this context, oral swabs represent a valuable non-invasive alternative when lymph node sampling is not feasible, particularly for identifying dogs requiring treatment [1].

Blood samples showed low sensitivity, in agreement with previous studies reporting low and intermittent parasitemia in CanL [41, 42], likely because blood is not a target tissue for parasite multiplication but rather a transport medium for dissemination [12, 41]. Additionally, the presence of PCR inhibitors may further reduce the sensitivity depending on the extraction protocol [43, 44]. For these reasons, blood should not be considered the sample of choice for molecular diagnosis of CanL [45]. However, despite this limitation, blood PCR showed certain stage-dependent discriminatory capacity, with nPCR differentiating early from advanced stages, while qPCR maintained discrimination for LV–III dogs in our study. Pantaleo et al. [46] also reported similar findings and, interestingly, a positive blood qPCR at diagnosis was predictive of persistent parasite detection in bone marrow after treatment, suggesting an association with an increased risk of relapse. Taken together, although blood is not suitable for routine molecular diagnosis, a positive qPCR result may identify dogs with more advanced disease and potentially lead to poorer treatment outcomes.

Urine has been proposed as a potential sample for the molecular detection of *L. infantum*, with several studies reporting relatively high PCR positivity and parasite loads mainly restricted to LV III–IV dogs [15, 46–48], as well as association with creatinine, symmetric dimethylarginine (SDMA) and proteinuria, supporting urine qPCR as a potential indicator of renal involvement and disease severity in CanL [15, 47]. By contrast, in our study, urine showed limited utility for the molecular detection of *L. infantum*, with very low positivity and parasite loads. These discrepancies between studies are likely related to methodological differences in DNA extraction protocols, as urine (particularly first-morning free-catch samples) may contain PCR inhibitors and bacterial endonucleases that can degrade DNA and compromise detection efficiency [49, 50]. In addition, the reduced specificity observed with urine qPCR may be explained by low-level non-specific amplification of environmental DNA, potentially originating from the diverse microbial DNA present in these samples, rather than true parasite detection. Future studies should focus on optimising and standardising DNA extraction from this matrix, incorporating steps to remove PCR inhibitors and improve assay sensitivity. Despite this overall low performance, the few nPCR-positive urine samples were also observed in dogs with more advanced clinical stages, but their low number did not allow any association between urine PCR and renal involvement. Only one LV–II dog tested positive by nPCR before treatment; this animal was an intact male, raising the possibility that the detected parasite DNA originated from residual seminal material [51] rather than renal excretion.

Similarly, hair samples showed very low sensitivity by nPCR in our study, with sporadic positive results restricted to dogs in advanced stages, although several studies reported the detection of *L. infantum* DNA in canine hair with sensitivities ranging from approximately 59–80% [16, 52–54], which may be partly explained by methodological differences, particularly the frequent use of kinetoplast DNA (kDNA) as PCR targets, which are generally more sensitive than genomic targets [45]. In this study, qPCR results from hair were excluded from the analysis owing to unreliable amplification patterns, likely caused by interference from dithiothreitol (DTT) used during DNA extraction, which can affect the fluorescent dyes used in qPCR and compromise DNA quantification [55, 56]. These artefacts were not observed in nPCR from the same DNA extracts, which supports the explanation of a technique-specific inhibition. Hair sampling is non-invasive and thus advantageous compared with lymph node or bone marrow, but presents intrinsic limitations such as low and variable parasite loads [16, 54], moderate reproducibility owing to inter-operator variability and potential co-amplification of non-*Leishmania* trypanosomatids [52], limiting its reliability for routine clinical use. Consequently, hair sampling may be more useful for epidemiological surveillance, particularly in settings where invasive sampling is impractical, such as wildlife monitoring in endemic areas [57, 58].

When comparing techniques, qPCR showed slightly higher sensitivity than nPCR in most tissues, except for lymph node and blood samples, where nPCR seems to perform better. Agreement (*κ*) between both techniques was almost perfect in LN, indicating that qPCR performs most reliably in tissues with higher parasite burdens. When agreement between non-invasive samples and LN was compared across techniques, differences reflected a complex interplay between technical sensitivity and the biological distribution of *Leishmania* across tissues. In OS, both techniques showed identical sensitivity, yet qPCR obtained higher agreement with LN, suggesting that qPCR positive results in oral swabs are more closely associated with systemic infection detectable in the lymph node. In CS, the lower agreement of qPCR with LN may be explained by the practical limitations of lymph node sampling, particularly in early stages, where qPCR may detect parasite DNA in conjunctival tissue while LN yields a negative result. As nPCR is less sensitive in CS, it tends to obtain concordant negative results in both tissues. In blood, the higher sensitivity and agreement of nPCR with LN may be partly explained by the greater robustness of nPCR against PCR inhibitors commonly present in this matrix, allowing more reliable detection of systemic infection.

Regarding treatment monitoring, dogs with clinically relevant disease showed a clear clinical improvement, with reductions in total scores, PCR positivity and parasite load, as shown in previous studies [25, 46, 59], particularly in LN, OS and CS. By contrast, IFAT titres remained unchanged at 30 days post-treatment, consistent with previous evidence indicating that serological titres decline slowly and are not suitable for early assessment of treatment efficacy [59]. In this context, qPCR from LN or OS appears to be a more sensitive indicator of early treatment response than serology. Blood and urine showed minimal changes after treatment, reinforcing their limited value for monitoring parasite dynamics owing to their low and intermittent parasite burden. The absence of differences in parasite load reduction between LV–II and LV–III dogs suggests that once generalised infection is established, early parasitological responses to treatment are comparable, regardless of initial clinical stage. Thus, although PCR represents a valuable complementary tool for monitoring parasitological response, therapeutic evaluation should primarily rely on clinical and laboratory improvement. In this sense, in our study, although clinical scores similarly improved in LV–II and LV–III dogs following treatment, only LV–II dogs showed significant improvement in laboratory parameters, highlighting the added value of the laboratory scoring system in detecting subtle stage-dependent differences in treatment response that may not be captured by changes in parasite load alone.

Despite marked parasite load reductions, PCR positivity persisted in some lymph node samples, reflecting the well-recognised ability of *Leishmania* to persist in tissues despite effective therapy [5, 6]. Conversely, PCR negativity should not be interpreted as parasitological cure, but rather as a reduction of parasite burden below the detection limit of the assay. This is supported by our cross-sectional findings at V1, where early-stage dogs showed very low PCR positivity despite previous confirmed infection. Following treatment, dogs may therefore shift to early LeishVet stages and remain clinically stable while harbouring residual parasites that remain undetectable by current molecular methods. This highlights the potential value of molecular techniques in long-term follow-up, as increases in parasite load may precede clinical relapse [5]. Indeed, while these results represent early post-treatment parasitological reduction, they are unlikely to reflect the full complexity of parasite dynamics in a chronic disease with a well-recognised relapse potential, highlighting the need for longer follow-up periods to better characterise molecular responses over time and assess long-term outcomes.

These findings demonstrate that the diagnostic and clinical value of PCR in CanL depends strongly on tissue selection, molecular technique and clinical stage. When integrated into a multimodal diagnostic approach combining clinical signs, clinicopathological, serological and parasitological data, PCR provides meaningful complementary information for identifying clinically relevant disease and monitoring parasite dynamics over time, particularly when invasive sampling is not feasible.

## Conclusions

PCR performance in CanL is strongly influenced by tissue type, molecular technique and clinical stage, emphasising that molecular results must be interpreted according to the specific clinical context. For initial diagnosis, nPCR should be the test of choice, as it proved equal or superior in sensitivity in three of the evaluated tissues, making it preferable for diagnostic confirmation of the infection in cryptic cases. Lymph node sampling remains the gold standard tissue for molecular confirmation of infection, providing the highest sensitivity and most consistent correlation with clinical disease, while oral swabs represent the most reliable non-invasive alternative when lymph node sampling is not feasible, although their diagnostic performance is mainly limited to dogs with moderate to severe disease (LeishVet II–III). Conjunctival swabs may serve as a secondary non-invasive option. Although blood PCR showed limited sensitivity for routine diagnosis, a positive result by nPCR may be associated with advanced clinical stages. For clinical follow-up, qPCR from lymph node or oral swabs is preferred, as it allows parasite load quantification and monitoring of treatment response through parasite dynamics. Technical challenges, such as PCR inhibition in hair samples and non-specific amplification in urine, limit the routine use of these matrices with the current protocol. PCR from non-invasive samples showed limited reliability in early stages of infection, reinforcing that molecular testing should complement, rather than replace, clinical evaluation, routine laboratory techniques and serology. Overall, these results support a multimodal diagnostic approach in which PCR is strategically applied according to tissue type, clinical stage and diagnostic purpose, supporting decision-making in both the diagnosis and long-term management of canine leishmaniosis.

## Supplementary Information


Additional file 1.Additional file 2.Additional file 3.Additional file 4.

## Data Availability

Data supporting the main conclusions of this study are included in the manuscript.

## Abbreviations

ALT: Alanine aminotransferase
AUC: Area under the curve
BEC: Blank extraction control
B: Blood
CanL: Canine leishmaniosis
CBC: Complete blood count
CLS: Clinical scoring
CS: Conjunctival swab
DTT: 1,4-Dithiothreitol
H: Hair
IFAT: Immunofluorescence antibody test
ICH: Infected clinically healthy
IRIS: International Renal Interest Society
LN: Lymph node
LOD: Limit of detection
LS: Laboratory scoring
LV–I: LeishVet stage I
LV–II: LeishVet stage II
LV–III: LeishVet stage III
LV–IV: LeishVet stage IV
MGA: Meglumine antimoniate
nPCR: Nested polymerase chain reaction
NI: Non-infected
NTC: No-template control
OS: Oral swab
PCR: Polymerase chain reaction
qPCR: Quantitative polymerase chain reaction
SDMA: Symmetric dimethylarginine
SSU rRNA: Small subunit ribosomal RNA
U: Urine
UPC: Urine protein/creatinine ratio

## References

[1] Solano-Gallego L, Koutinas A, Miró G, Cardoso L, Pennisi MG, Ferrer L, et al. Directions for the diagnosis, clinical staging, treatment and prevention of canine leishmaniosis. Vet Parasitol. 2009;165:1–18. 10.1016/j.vetpar.2009.05.022. 19559536

[2] Gramiccia M, Gradoni L. The current status of zoonotic leishmaniases and approaches to disease control. Int J Parasitol. 2005;35:1169–80. 10.1016/j.ijpara.2005.07.001. 16162348

[3] Miró G, Montoya A, Mateo M, Alonso A, García S, García A, et al. A leishmaniosis surveillance system among stray dogs in the region of Madrid: ten years of serodiagnosis (1996–2006). Parasitol Res. 2007;101:253–7. 10.1007/s00436-007-0497-8. 17323100

[4] Bourdeau P, Saridomichelakis MN, Oliveira A, Oliva G, Kotnik T, Gálvez R, et al. Management of canine leishmaniosis in endemic SW European regions: a questionnaire-based multinational survey. Parasit Vectors. 2014;7:110. 10.1186/1756-3305-7-110. 24656172 PMC3974741

[5] Manna L, Reale S, Vitale F, Picillo E, Pavone LM, Gravino AE. Real-time PCR assay in *Leishmania*-infected dogs treated with meglumine antimoniate and allopurinol. Vet J. 2008;177:279–82. 10.1016/j.tvjl.2007.04.013. 17553711

[6] Baneth G, Shaw SE. Chemotherapy of canine leishmaniosis. Vet Parasitol. 2002;106:315–24. 10.1016/S0304-4017(02)00115-2. 12079737

[7] Paltrinieri S, Solano-Gallego L, Fondati A, Lubas G, Gradoni L, Castagnaro M, et al. Guidelines for diagnosis and clinical classification of leishmaniasis in dogs. J Am Vet Med Assoc. 2010;236:1184–91. 10.2460/javma.236.11.1184. 20513195

[8] Maia C, Campino L. Methods for diagnosis of canine leishmaniasis and immune response to infection. Vet Parasitol. 2008;158:274–87. 10.1016/j.vetpar.2008.07.028. 18789583

[9] Manna L, Reale S, Viola E, Vitale F, Manzillo VF, Michele PL, et al. *Leishmania* DNA load and cytokine expression levels in asymptomatic naturally infected dogs. Vet Parasitol. 2006;142:271–80. 10.1016/j.vetpar.2006.06.028. 16920264

[10] De Almeida Ferreira S, Leite RS, Ituassu LT, Almeida GG, Souza DM, Fujiwara RT, et al. Canine skin and conjunctival swab samples for the detection and quantification of *Leishmania infantum* DNA in an endemic urban area in Brazil. PLoS Negl Trop Dis. 2012;6:e1596. 10.1371/journal.pntd.0001596. 22506084 PMC3323509

[11] Strauss‐Ayali D, Jaffe CL, Burshtain O, Gonen L, Baneth G. Polymerase chain reaction using noninvasively obtained samples, for the detection of *Leishmania infantum* DNA in dogs. J Infect Dis. 2004;189:1729–33. 10.1086/383281. 15116312

[12] Hernández L, Montoya A, Checa R, Dado D, Gálvez R, Otranto D, et al. Course of experimental infection of canine leishmaniosis: follow-up and utility of noninvasive diagnostic techniques. Vet Parasitol. 2015;207:149–55. 10.1016/j.vetpar.2014.10.035. 25692190

[13] Ferreira SdeA, Almeida GG, Silva SdeO, Vogas GP, Fujiwara RT, Andrade ASRde, et al. Nasal, oral and ear swabs for canine visceral leishmaniasis diagnosis: new practical approaches for detection of *Leishmania infantum* DNA. PLoS Negl Trop Dis. 2013;7:e2150. 10.1371/journal.pntd.0002150. 23593518 PMC3617150

[14] Carvalho Ferreira AL, Carregal VM, de Almeida Ferreira S, Leite RS, de Andrade ASR. Detection of *Leishmania infantum* in 4 different dog samples by real-time PCR and ITS-1 nested PCR. Diagn Microbiol Infect Dis. 2014;78:418–21. 10.1016/j.diagmicrobio.2013.10.015. 24485588

[15] Solano-Gallego L, Rodriguez-Cortes A, Trotta M, Zampieron C, Razia L, Furlanello T, et al. Detection of *Leishmania infantum* DNA by FRET-based real-time PCR in urine from dogs with natural clinical leishmaniosis. Vet Parasitol. 2007;147:315–9. 10.1016/j.vetpar.2007.04.013. 17532143

[16] Belinchón-Lorenzo S, Iniesta V, Parejo JC, Fernández-Cotrina J, Muñoz-Madrid R, Soto M, et al. Detection of *Leishmania infantum* kinetoplast minicircle DNA by real time PCR in hair of dogs with leishmaniosis. Vet Parasitol. 2013;192:43–50. 10.1016/j.vetpar.2012.11.007. 23218222

[17] Di Muccio T, Veronesi F, Antognoni MT, Onofri A, Piergili Fioretti D, Gramiccia M. Diagnostic value of conjunctival swab sampling associated with nested PCR for different categories of dogs naturally exposed to *Leishmania infantum* infection. J Clin Microbiol. 2012;50:2651–9. 10.1128/JCM.00558-12. 22649018 PMC3421491

[18] Cavalera MA, Zatelli A, Donghia R, Mendoza-Roldan JA, Gernone F, Otranto D, et al. Conjunctival swab real time-PCR in *Leishmania infantum* seropositive dogs: diagnostic and prognostic values. Biology. 2022;11:184. 10.3390/biology11020184. 35205050 PMC8869220

[19] Fernandes MA, Leonel JAF, Isaac JA, Benassi JC, Silva DT, Spada JCP, et al. Molecular detection of *Leishmania infantum* DNA according to clinical stages of leishmaniasis in dog. Rev Bras Parasitol Vet. 2019;28:194–202. 10.1590/s1984-29612019015. 31188942

[20] Peris MP, Esteban-Gil A, Ortega-Hernández P, Morales M, Halaihel N, Castillo JA. Comparative study of real-time PCR (TaqMan Probe and Sybr Green), serological techniques (ELISA, IFAT and DAT) and clinical signs evaluation, for the diagnosis of canine leishmaniasis in experimentally infected dogs. Microorganisms. 2021;9:2627. 10.3390/microorganisms9122627. 34946227 PMC8704913

[21] Chicharro C, Nieto J, Miguelañez S, Garcia E, Ortega S, Peña A, et al. Molecular diagnosis of leishmaniasis in Spain: development and validation of ready-to-use gel-form nested and real-time PCRs to detect *Leishmania* spp. Microbiol Spectr. 2023;11:e0335422. 10.1128/spectrum.03354-22. 37014253 PMC10269443

[22] Cruz I, Millet A, Carrillo E, Chenik M, Salotra P, Verma S, et al. An approach for interlaboratory comparison of conventional and real-time PCR assays for diagnosis of human leishmaniasis. Exp Parasitol. 2013;134:281–9. 10.1016/j.exppara.2013.03.026. 23562705

[23] León CM, Muñoz M, Hernández C, Ayala MS, Flórez C, Teherán A, et al. Analytical performance of four polymerase chain reaction (PCR) and real time PCR (qPCR) assays for the detection of six *Leishmania* species DNA in Colombia. Front Microbiol. 2017;8:1907. 10.3389/fmicb.2017.01907. 29046670 PMC5632848

[24] Cruz I, Cañavate C, Rubio JM, Morales MA, Chicharro C, Laguna F, et al. A nested polymerase chain reaction (Ln-PCR) for diagnosing and monitoring *Leishmania infanturn* infection in patients co-infected with human immuno-deficiency virus. Trans R Soc Trop Med Hyg. 2002;96:5. 10.1016/s0035-9203(02)90074-x 12055836

[25] Miró G, Oliva G, Cruz I, Cañavate C, Mortarino M, Vischer C, et al. Multicentric, controlled clinical study to evaluate effectiveness and safety of miltefosine and allopurinol for canine leishmaniosis. Vet Dermatol. 2009;20:397–404. 10.1111/j.1365-3164.2009.00824.x. 20178476

[26] Mancianti F, Meciani N. Specific serodiagnosis of canine leishmaniasis by indirect immunofluorescence, indirect hemagglutination, and counterimmunoelectrophoresis. Am J Vet Res. 1988;49:1409–11. 3052194

[27] Sarquis J, Parody N, Montoya A, Cacheiro-Llaguno C, Barrera JP, Checa R, et al. Clinical validation of circulating immune complexes for use as a diagnostic marker of canine leishmaniosis. Front Vet Sci. 2024. 10.3389/fvets.2024.1368929. 38562919 PMC10984162

[28] IRIS Guidelines. International Renal Interest Society (IRIS). 2022. https://www.iris-kidney.com/iris-guidelines-1.

[29] Van Eys GJJM, Schoone GJ, Kroon NCM, Ebeling SB. Sequence analysis of small subunit ribosomal RNA genes and its use for detection and identification of *Leishmania* parasites. Mol Biochem Parasitol. 1992;51:133–42. 10.1016/0166-6851(92)90208-2. 1565128

[30] Norman FF, Pérez-Ayala A, Pérez-Molina JA, Flores-Chavez M, Cañavate C, López-Vélez R. Lack of association between blood-based detection of *Trypanosoma cruzi* DNA and cardiac involvement in a non-endemic area. Ann Trop Med Parasitol. 2011;105:425–30. 10.1179/1364859411Y.0000000033. 22117851 PMC4100305

[31] Almeida ABPF, Sousa VRF, Gasparetto ND, da Silva GFR, Figueiredo FB, Dutra V, et al. Canine visceral leishmaniasis: diagnostic approaches based on polymerase chain reaction employing different biological samples. Diagn Microbiol Infect Dis. 2013;76:321–4. 10.1016/j.diagmicrobio.2013.03.017. 23619344

[32] Momo C, Jacintho APP, Moreira PRR, Munari DP, Machado GF, Vasconcelos RdeO. Morphological changes in the bone marrow of the dogs with visceral leishmaniasis. Vet Med Int. 2014;2014:1–5. 10.1155/2014/150582. PMC3972870 24744957

[33] Reithinger R, Lambson BE, Barker DC, Counihan H, Espinoza JC, Gonzalez JS, et al. *Leishmania *(*Viannia*)* spp.* dissemination and tissue tropism in naturally infected dogs (*Canis familiaris*). Trans R Soc Trop Med Hyg. 2002;96:76–8. 10.1016/S0035-9203(02)90249-X. 11926001

[34] Aslan H, Oliveira F, Meneses C, Castrovinci P, Gomes R, Teixeira C, et al. New insights into the transmissibility of *Leishmania infantum* from dogs to sand flies: experimental vector-transmission reveals persistent parasite depots at bite sites. J Infect Dis. 2016;213:1752–61. 10.1093/infdis/jiw022. 26768257 PMC4857470

[35] Lombardo G, Pennisi MG, Pennisi MG, Lupo T, Lupo T, Migliazzo A, et al. Detection of *Leishmania infantum* DNA by real-time PCR in canine oral and conjunctival swabs and comparison with other diagnostic techniques. Vet Parasitol. 2012;184:10–7. 10.1016/j.vetpar.2011.08.010. 21906883

[36] Ceccarelli M, Galluzzi L, Sisti D, Bianchi B, Magnani M. Application of qPCR in conjunctival swab samples for the evaluation of canine leishmaniasis in borderline cases or disease relapse and correlation with clinical parameters. Parasit Vectors. 2014;7:460. 10.1186/s13071-014-0460-3. 25331737 PMC4207623

[37] Leite RS, Souza NA, Barbosa AD, Ferreira ALC, De Andrade ASR. Evaluation of conjunctival swab as a mass-screening tool for molecular diagnosis of canine visceral leishmaniasis. Parasitol Res. 2015;114:2255–62. 10.1007/s00436-015-4418-y. 25782681

[38] Sarquis J, Raposo LM, Sanz CR, Montoya A, Barrera JP, Checa R, et al. Relapses in canine leishmaniosis: risk factors identified through mixed-effects logistic regression. Parasit Vectors. 2024;17:357. 10.1186/s13071-024-06423-1. 39175031 PMC11342489

[39] Manna L, Reale S, Vitale F, Gravino AE. Evidence for a relationship between *Leishmania* load and clinical manifestations. Res Vet Sci. 2009;87:76–8. 10.1016/j.rvsc.2008.12.009. 19178919

[40] Chagas ÚMR, de Avelar DM, Marcelino AP, Paz GF, Gontijo CMF. Correlations between tissue parasite load and common clinical signs in dogs naturally infected by *Leishmania infantum*. Vet Parasitol. 2021;291:109368. 10.1016/j.vetpar.2021.109368. 33556846

[41] Di Pietro S, Crinò C, Falcone A, Crupi R, Francaviglia F, Vitale F, et al. Parasitemia and its daily variation in canine leishmaniasis. Parasitol Res. 2020;119:3541–8. 10.1007/s00436-020-06845-7. 32803333 PMC7429123

[42] Maia C, Cristóvão J, Pereira A, Kostalova T, Lestinova T, Sumova P, et al. Monitoring *Leishmania* infection and exposure to *Phlebotomus perniciosus* using minimal and non-invasive canine samples. Parasit Vectors. 2020;13:119. 10.1186/s13071-020-3993-7. 32312325 PMC7171869

[43] Lachaud L, Chabbert E, Dubessay P, Reynes J, Lamothe J, Bastien P. Comparison of various sample preparation methods for PCR diagnosis of visceral leishmaniasis using peripheral blood. J Clin Microbiol. 2001;39:613–7. 10.1128/JCM.39.2.613-617.2001. 11158116 PMC87785

[44] Lachaud L, Marchergui-Hammami S, Chabbert E, Dereure J, Dedet JP, Bastien P. Comparison of six PCR methods using peripheral blood for detection of canine visceral leishmaniasis. J Clin Microbiol. 2002;40:210–5. 10.1128/jcm.40.1.210-215.2002. 11773118 PMC120090

[45] Miró G, Cardoso L, Pennisi MG, Oliva G, Baneth G. Canine leishmaniosis—new concepts and insights on an expanding zoonosis: Part Two. Trends Parasitol. 2008;24:371–7. 10.1016/j.pt.2008.05.003. 18603476

[46] Pantaleo V, Furlanello T, Campigli M, Ventura L, Solano-Gallego L. Short term treatment monitoring of renal and inflammatory biomarkers with naturally occurring leishmaniosis: a cohort study of 30 dogs. Vet Sci. 2024;11:517. 10.3390/vetsci11110517. 39591291 PMC11598865

[47] Manna L, Reale S, Picillo E, Vitale F, Gravino AE. Urine sampling for real-time polymerase chain reaction-based diagnosis of canine leishmaniasis. J Vet Diagn Invest. 2008;20:64–7. 10.1177/104063870802000112. 18182511

[48] Franceschi A, Merildi V, Guidi G, Mancianti F. Occurrence of *Leishmania* DNA in urines of dogs naturally infected with leishmaniasis. Vet Res Commun. 2007;31:335–41. 10.1007/s11259-006-3477-z. 17186402

[49] Silva MALD, Medeiros Z, Soares CRP, Silva EDD, Miranda-Filho DB, Melo FLD. A comparison of four DNA extraction protocols for the analysis of urine from patients with visceral leishmaniasis. Rev Soc Bras Med Trop. 2014;47:193–7. 10.1590/0037-8682-0233-2013. 24861293

[50] Bezerra GSN, Barbosa WL, Silva EDD, Leal NC, Medeiros ZMD. Urine as a promising sample for *Leishmania* DNA extraction in the diagnosis of visceral leishmaniasis—a review. Braz J Infect Dis. 2019;23:111–20. 10.1016/j.bjid.2019.04.001. 31054271 PMC9425670

[51] Diniz SA, Melo MS, Borges AM, Bueno R, Reis BP, Tafuri WL, et al. Genital lesions associated with visceral leishmaniasis and shedding of *Leishmania spp.* in the semen of naturally infected dogs. Vet Pathol. 2005. 10.1354/vp.42-5-650. 16145211

[52] Vioti G, Leonel JAF, De Sousa Oliveira TMF, Da Silva DT, Alves ML, Nakaghi ACH, et al. Evaluation of hair samples for PCR detection of *Leishmania infantum* in dogs. Res Vet Sci. 2026;198:105979. 10.1016/j.rvsc.2025.105979. 41297444

[53] De Sousa Gonçalves R, Franke CR, Magalhães-Junior JT, Souza BMPS, Solcà MS, Larangeira DF, et al. Association between *Leishmania infantum* DNA in the hair of dogs and their infectiousness to *Lutzomyia longipalpis*. Vet Parasitol. 2016;232:43–7. 10.1016/j.vetpar.2016.11.010. 27890081

[54] Corpas-López V, Merino-Espinosa G, Acedo-Sánchez C, Díaz-Sáez V, Morillas-Márquez F, Martín-Sánchez J. Hair parasite load as a new biomarker for monitoring treatment response in canine leishmaniasis. Vet Parasitol. 2016;223:20–5. 10.1016/j.vetpar.2016.04.001. 27198771

[55] Hudson BC, Cox JO, Seashols-Williams SJ, Dawson Cruz T. The effects of dithiothreitol (DTT) on fluorescent qPCR dyes. J Forensic Sci. 2021;66:700–8. 10.1111/1556-4029.14637. 33284476

[56] Boiso L, Sanga M, Hedman J. DTT quenches the passive reference signal in real-time PCR. Forensic Sci Int Genet Suppl. 2015;5:e5–6. 10.1016/j.fsigss.2015.09.003.

[57] Muñoz-Madrid R, Belinchón-Lorenzo S, Iniesta V, Fernández-Cotrina J, Parejo JC, Serrano FJ, et al. First detection of *Leishmania infantum* kinetoplast DNA in hair of wild mammals: application of qPCR method to determine potential parasite reservoirs. Acta Trop. 2013;128:706–9. 10.1016/j.actatropica.2013.08.009. 23973736

[58] Ortega MV, Moreno I, Domínguez M, de la Cruz ML, Martín AB, Rodríguez-Bertos A, et al. Application of a specific quantitative real-time PCR (qPCR) to identify *Leishmania infantum* DNA in spleen, skin and hair samples of wild *Leporidae*. Vet Parasitol. 2017;243:92–9. 10.1016/j.vetpar.2017.05.015. 28807318

[59] Mateo M, Maynard L, Vischer C, Bianciardi P, Miró G. Comparative study on the short-term efficacy and adverse effects of miltefosine and meglumine antimoniate in dogs with natural leishmaniosis. Parasitol Res. 2009;105:155–62. 10.1007/s00436-009-1375-3. 19238439

