# Supplementary material for: Validation of a duplex real-time PCR (qPCR) in clinically classified dogs naturally infected with Leishmania infantum before and after specific treatment: preliminary results
Source: Parasit Vectors. 2026 Jun 21;19:374. doi: 10.1186/s13071-026-07490-2 (PMC13531858; doi:10.1186/s13071-026-07490-2)
Supplement: Supplementary file 1 [file 13071_2026_7490_MOESM1_ESM.docx]

Additional file 1: Table S1. Clinical scoring system used in this study.

| Clinical condition/ affected organ | Clinical sign | | Score | | | |
| --- | --- | --- | --- | --- | --- | --- |
|  |  |  | 0 | 1 | 2 | 3 |
| Systemic | Appetite | | Normal | Dysorexia | Hyporexia | Anorexia |
|  | Lethargy | | Absent | Mild | Severe | - |
|  | Weight loss | | Absent | Mild (<10%) | Moderate (10-20%) | Severe (>20%) |
|  | Fever | | Absent | - | Present | - |
| Musculature | Muscle atrophy | | Absent | Moderate | - | Sarcopenia |
| Mononuclear phagocytic system | Lymnphadenomegaly | | Absent | Localised | - | Generalised |
|  | Hepatomegaly | | Absent | - | - | Present |
|  | Splenomegaly | | Absent | - | - | Present |
| Ocular manifestations | Conjunctivitis | | Absent | Present | - | - |
|  | Blepharitis | | Absent | Present | - | - |
|  | Keratitis | | Absent | - | Present | - |
| Mucous membranes | Pale mucous membranes | | Absent | - | Present | - |
|  | Oral ulcers | | Absent | Single/Focal | Multiple | - |
| Digestive system | Vomiting | | Absent | Occasional | Persistent/haemorrhagic | - |
|  | Diarrhoea | | Absent | Occasional | Persistent/haemorrhagic | - |
| Nervous system | Paresis/paralysis | | Absent | - | Paraparesis | Tetraparesis or para-/tetraplegia |
| Immune-complex deposition | Polyuria/ Polydipsia | | Absent | - | Present | - |
|  | Epistaxis | | Absent | Occasional bleeding | Frequent bleeding | Uncontrollable bleeding |
|  | Vasculitis | | Absent | - | Moderate | Severe |
|  | Uveitis | | Absent | Mild, resolves with topical treatment | Moderate, requires oral treatment | Severe, risk of blindness |
|  | Lameness and/or joint pain | | Absent | Mild (no effect on movement) | Moderate (mild discomfort) | Severe lameness affecting mobility |
| Cutaneous disorders | Papular dermatitis | | Absent | Single papule | Multiple papules | - |
|  | Exfoliative dermatitis | Alopecia | Absent | Localised_a_ or mild | Generalised | - |
|  |  | Dry seborrhoea | Absent | Localised_a_ or mild | Generalised | - |
|  |  | Oily seborrhoea | Absent | Localised_a_ or mild | Generalised | - |
|  |  | Hyperpigmentation | Absent | Localised^a^ or mild | Generalised | - |
|  |  | Hypopigmentation | Absent | Localised_a_ or mild | Generalised | - |
|  |  | Hyperkeratosis | Absent | Localised_a_ or mild | Generalised | - |
|  | Ulcerative dermatitis | | Absent | Single ulcer | Multiple ulcers | - |
|  | Nodular dermatitis | | Absent | Single nodule | Multiple nodules | - |
|  | Onychogryphosis | | Absent | - | Present | - |
|  | Other | | Absent | Present | - | - |
| Total score |  | | | | | __/70 |

_a_Localised: <10% of body surface
