# Supplementary material for: Validation of a duplex real-time PCR (qPCR) in clinically classified dogs naturally infected with Leishmania infantum before and after specific treatment: preliminary results
Source: Parasit Vectors. 2026 Jun 21;19:374. doi: 10.1186/s13071-026-07490-2 (PMC13531858; doi:10.1186/s13071-026-07490-2)
Supplement: Supplementary file 2 [file 13071_2026_7490_MOESM2_ESM.docx]

Additional file 2: Table S2. Scoring system for laboratory abnormalities used in this study.

| Laboratory abnormalities | | | 0 | 1 | 2 | 3 |
| --- | --- | --- | --- | --- | --- | --- |
| Complete Blood Count | Haematocrit (%) | | Normal | Mild anaemia (30-35%) | Moderate anaemia (20-30%) | Severe anaemia (<20%) |
|  | Leukocytes (x10^3^/µl) | Leukocytosis | Absent | - | Moderate (14.1-16.0 x 10^3^) | Severe (>16.0 x 10^3^) |
|  |  | Leukopenia | Absent | - | Moderate (4.0 - 5.0 x 10^3^) | Severe (< 4.0 x 10^3^) |
|  | Platelet count (x10^3^/µl) | Thrombocytopathy | Absent | - | Moderate (500 - 600 x 10^3^) | Severe (> 600 x 10^3^) |
|  |  | Thrombocytopenia | Absent | - | Moderate (80 - 200 x 10^3^) | Severe (< 80 x 10^3^) |
|  | Blood smear | | Normal | Altered _a_ | - | - |
| Serum biochemical profile | Urea (mg/dl) | | Normal | - | Slightly increased (58 - 100 mg/dl) | Severely increased (> 100 mg/dl) |
|  | Creatinine (mg/dl) | | Normal | - | Slightly increased (1.4 - 2.8 mg/dl) | Severely increased (> 2.8 mg/dl) |
|  | Alanine Aminotransferase (U/l) | | Normal | - | Slightly increased (60 - 125 U/L) | Severely increased (> 125 U/L) |
| Serum protein electrophoresis | Total proteins (g/dl) | | Normal | - | Moderately increased (7.5 - 9.0 g/dl) | Severely increased (> 9.0 g/dl) |
|  | Albumin (g/dl) | | Normal | - | Decreased (2.0 - 2.4 g/dl) | Severely decreased (< 2.0 g/dl) |
|  | Albumin/Globulin ratio | | Normal | - | Decreased (0.5 - 0.7) | Severely decreased (< 0.5) |
|  | Alpha 1 Globulins (g/dl) | | Normal | - | Increased (> 0.5 g/dl) | - |
|  | Alpha 2 Globulins (g/dl) | | Normal | - | Increased (> 1.1 g/dl) | - |
|  | Beta Globulins (g/dl) | | Normal | - | Increased (2.7 - 4.0 g/dl) | Severely increased (> 4.0 g/dl) |
|  | Gamma Globulins (g/dl) | | Normal | Slightly increased (1.2 - 2.0 g/dl) | Increased (2.0 - 4.0 g/dl) | Severely increased (> 4.0 g/dl) |
| Urianalysis | Urine specific gravity | | Normal (> 1,030) | Inadequate concentration (1.013 - 1.029) | Isosthenuria (1,008 - 1,012) | Hyposthenuria (< 1,008) |
|  | Urine protein/creatinine ratio | | Normal (< 0.2) | Mild proteinuria (0.2 - 0.5) | Moderate proteinuria (0.5 - 1.0) | Severe proteinuria (> 1.0) |
|  | Cylindruria (specify type) | | Absent | - | Present | - |
| Total score |  | | | | | __/50 |

_a_ Altered blood smear: Presence of erythrocyte rouleaux, changes in leukocyte morphology, other (specify)

Cut-off values were defined according to the reference intervals established by the clinical pathology laboratory of the Veterinary Teaching Hospital, Complutense University of Madrid.
