# Supplementary material for: Validation of a duplex real-time PCR (qPCR) in clinically classified dogs naturally infected with Leishmania infantum before and after specific treatment: preliminary results
Source: Parasit Vectors. 2026 Jun 21;19:374. doi: 10.1186/s13071-026-07490-2 (PMC13531858; doi:10.1186/s13071-026-07490-2)
Supplement: Supplementary file 3 [file 13071_2026_7490_MOESM3_ESM.docx]

Additional file 3: Table S3. Clinical data and laboratory parameters across groups at Visit 1.

| Variable | Reference interval | | NI (n = 20) | ICH (n = 15) | LV-I (n = 7) | LV-II (n = 14) | LV-III (n = 10) | Total (n = 66) | *P* value (across groups) |
| --- | --- | --- | --- | --- | --- | --- | --- | --- | --- |
|  |  | | Mean score ± SD | | | | | |  |
| Clinical score | - | | 0.80 ± 1.20 | 0.87 ± 1.64 | 3.14 ± 2.04 | 9.00 ± 7.85 | 11.50 ± 4.77 | 4.42 ± 6.02 | < 0.001 |
| Laboratory score | - | | 3.30 ± 1.92 | 2.33 ± 2.74 | 3.14 ± 1.68 | 10.21 ± 6.40 | 13.70 ± 6.18 | 6.11 ± 5.97 | < 0.001 |
|  | | | % dogs (n) | | | | | |  |
| Systemic signs_a_ | | - | 5.0 (1) | 13.3 (2) | 14.3 (1) | 71.4 (10) | 100.0 (10) | 36.4 (24) | < 0.001 |
| Cutaneous signs_b_ | | - | 20.0 (4) | 33.3 (5) | 71.4 (5) | 85.7 (12) | 100.0 (10) | 54.5 (36) | < 0.001 |
| Signs of immune-complex deposition_c_ | | - | 5.0 (1) | 0 (0) | 1.4 (1) | 28.6 (4) | 70.0 (7) | 19.7 (13) | < 0.001 |
|  |  | | Mean value ± SD | | | | | |  |
| Hct (%) | 35-57 | | 48.0 ± 4.5 | 51.8 ± 4.5 | 46.1 ± 3.5 | 36.9 ± 9.2 | 37.3 ± 11.3 | 44.7 ± 9.1 | < 0.001 |
| WBC (x10^3^/µl) | 5.0-14.1 | | 7.8 ± 2.4 | 8.9 ± 2.6 | 8.0 ± 2.0 | 9.7 ± 5.6 | 7.2 ± 3.3 | 8.4 ± 3.5 | 0.585 |
| Plt (x10^3^/µl) | 200-500 | | 321.5 ± 120.7 | 270.7 ± 87.1 | 260.0 ± 59.4 | 231.5 ± 89.0 | 167.9 ± 82.1 | 257.8 ± 107.2 | 0.011 |
| Urea (mg/dl) | 15-58 | | 34.1 ± 14.1 | 33.7 ± 11.9 | 26.3 ± 11.5 | 29.9 ± 11.8 | 37.2 ± 18.5 | 32.8 ± 13.6 | 0.630 |
| Creatinine (mg/dl) | 0.5-1.8 | | 0.9 ± 0.2 | 1.0 ± 0.2 | 1.1 ± 0.2 | 0.9 ± 0.2 | 1.1 ± 0.4 | 1.0 ± 0.3 | 0.081 |
| ALT (U/L) | 10-125 | | 89.3 ± 75.3 | 48.0 ± 17.6 | 38.1 ± 17.0 | 89.8 ± 197.7 | 54.4 ± 57.8 | 69.3 ± 102.4 | 0.005 |
| Prot (g/dl) | 5.8-7.5 | | 6.7 ± 0.4 | 6.5 ± 0.6 | 7.3 ± 0.5 | 7.7 ± 1.1 | 8.4 ± 1.8 | 7.2 ± 1.2 | < 0.001 |
| Alb (g/dl) | 2.4-3.9 | | 3.3 ± 0.4 | 3.3 ± 0.4 | 3.2 ± 0.2 | 2.4 ± 0.7 | 2.4 ± 0.4 | 3.0 ± 0.6 | < 0.001 |
| Alb/Glob ratio | 0.7-1.9 | | 1.0 ± 0.3 | 1.1 ± 0.3 | 0.8 ± 0.1 | 0.5 ± 0.2 | 0.4 ± 0.2 | 0.8 ± 0.4 | < 0.001 |
| Alpha 1 glob (g/dl) | 0.2-0.5 | | 0.3 ± 0.1 | 0.3 ± 0.1 | 0.3 ± 0.1 | 0.3 ± 0.1 | 0.3 ± 0.1 | 0.3 ± 0.1 | 0.410 |
| Alpha 2 glob (g/dl) | 0.3-1.1 | | 1.0 ± 0.3 | 0.8 ± 0.3 | 1.0 ± 0.1 | 0.9 ± 0.2 | 0.8 ± 0.3 | 0.9 ± 0.3 | 0.276 |
| Beta glob (g/dl) | 1.3-2.7 | | 1.5 ± 0.3 | 1.4 ± 0.4 | 1.8 ± 0.3 | 2.2 ± 0.7 | 1.9 ± 0.5 | 1.7 ± 0.5 | < 0.001 |
| Gamma glob (g/dl) | 0.5-1.2 | | 0.5 ± 0.1 | 0.6 ± 0.1 | 0.9 ± 0.2 | 1.9 ± 1.1 | 2.9 ± 1.9 | 1.2 ± 1.2 | < 0.001 |
| UPC ratio | <0.2 | | 0.1 ± 0.1 | 0.3 ± 0.6 | 0.1 ± 0.1 | 0.3 ± 0.3 | 1.4 ± 1.3 | 0.5 ± 0.8 | 0.002 |
|  | IFAT titer | | % dogs (n) | | | | | |  |
| IFAT | Negative (<1/200) | | 100.0 (20) | 66.7 (10) | 28.6 (2) | 0 (0) | 0 (0) | 48.5 (32) | < 0.001 |
|  | Low positive (1/200-1/400) | | 0 (0) | 33.3 (5) | 71.4 (5) | 14.3 (2) | 10.0 (1) | 19.7 (13) | < 0.001 |
|  | Medium positive (1/800-1/1600) | | 0 (0) | 0 (0) | 0 (0) | 35.7 (5) | 40.0 (4) | 13.6 (9) | 0.001 |
|  | High positive (> 1/1600) | | 0 (0) | 0 (0) | 0 (0) | 50.0 (7) | 50.0 (5) | 18.2 (12) | < 0.001 |

_a_Systemic signs: loss of appetite, lethargy, weight loss and/or fever; _b_cutaneous signs: papular dermatitis, exfoliative dermatitis, ulcerative dermatitis, nodular dermatitis, onychogryphosis; _c_signs of immune-complex deposition: polyuria/polydipsia, epistaxis, vasculitis, uveitis, lameness and/or joint pain. Reference intervals used to define laboratory abnormalities were those established by the clinical pathology laboratory of the Veterinary Teaching Hospital, Complutense University of Madrid. Abbreviations: Hct, haematocrit; WBC, white blood cells; Plt, platelets; ALT, alanine aminotransferase; Prot, total proteins; Alb, albumin; Glob, globulins; UPC, urine protein/creatinine ratio; SD, standard deviation.
