# Supplementary material for: Validation of a duplex real-time PCR (qPCR) in clinically classified dogs naturally infected with Leishmania infantum before and after specific treatment: preliminary results
Source: Parasit Vectors. 2026 Jun 21;19:374. doi: 10.1186/s13071-026-07490-2 (PMC13531858; doi:10.1186/s13071-026-07490-2)
Supplement: Supplementary file 4 [file 13071_2026_7490_MOESM4_ESM.docx]

Additional file 4: Table S4. Distribution of qPCR C_T_ values by tissue and clinical group at Visit 1.

|  | LN | CS | OS | B | U |
| --- | --- | --- | --- | --- | --- |
|  | Median C_T_ (IQR) | | | | |
| NI  (n = 20) | 45.00 | 45.00 | 45.00 | 45.00 | 45.00 (43.88 - 45.00) |
| ICH  (n = 15) | 45.00 (39.62-45.00) | 45.00 | 45.00 | 45.00 | 45.00 |
| LV-I  (n = 7) | 45.00 | 45.00 (38.21 - 45.00) | 45.00 | 45.00 (38.26 - 45.00) | 45.00 |
| LV-II  (n = 14) | 23.58 (22.01 - 32.04) | 37.38 (33.02 - 45.00) | 34.04 (31.42 - 36.10) | 45.00 (36.68 - 45.00) | 45.00 |
| LV-III  (n = 10) | 25.28 (24.03 - 29.62) | 37.86 (33.79 - 45.00) | 36.53 (32.91 - 45.00) | 39.34 (36.47 - 45.00) | 45.00 |

qPCR: quantitative PCR; NI: non-infected dogs; ICH: infected clinically healthy dogs; LV I-III: dogs classified as LeishVet stages I-III; n: number of dogs; LN: Lymph node; CS: conjunctival swabs; OS: oral swabs; B: blood; U: urine; IQR: Interquartile Range. CT values are expressed as median (IQR). Samples with no detectable amplification were assigned the maximum cycle value of the assay (C_T_ = 45.00) for analytical purposes. When IQR is not shown, ≥ 75% of samples did not yield amplification. C_T_ cut-off value for qPCR positivity was set at a C_T_ < 39.00 by receiver operating characteristic (ROC) curve analysis for each tissue with additional consideration of clinical applicability, using clinical classification as the reference standard.
